# Targeted-detection and sequential-treatment of small hepatocellular carcinoma in the complex liver environment by GPC-3-targeted nanoparticles

**DOI:** 10.1186/s12951-022-01378-w

**Published:** 2022-03-24

**Authors:** Han Deng, Wenting Shang, Kun Wang, Kunxiong Guo, Yu Liu, Jie Tian, Chihua Fang

**Affiliations:** 1 Department of Hepatobiliary Surgery, Institute for Digital Intelligence, Zhujiang Hospital, Southern Medical University, Guangzhou, 510280 China; 2 CAS Key Laboratory of Molecular Imaging, Beijing Key Laboratory of Molecular Imaging, the State Key Laboratory of Management and Control for Complex Systems, Institute of Automation, Chinese Academy of Sciences, Beijing, 100190 China; 3 Beijing Advanced Innovation Center for Big Data-Based Precision Medicine, School of Medicine and Engineering, Beihang University, Beijing, 100191 China; 4 Provincial Clinical and Engineering Center of Digital Medicine, Guangzhou, 510280 China

**Keywords:** Small hepatocellular carcinoma, Complex liver environment, Targeted detection, Dynamic contrast-enhanced photoacoustic imaging, Sequential catalysis-targeted therapy

## Abstract

Despite advancements in diagnostic methods and therapeutic strategies, the mortality rate of hepatocellular carcinoma (HCC) remains as high as its incidence rate. Most liver cancers are detected in the advanced stages, when treatment options are limited. Small HCC is difficult to diagnose and is often overlooked by current imaging methods because of the complexity of the liver environment, especially in cirrhotic livers. In the present study, we developed a tumor “cruise missile”, mesoporous Fe_3_O_4_-containing glucose oxidase-conjugated GPC3 peptide nanoparticles (FGP NPs). It was designed to enhance the accuracy of small HCC visualization to 85.7% using combined ultrasound/photoacoustic imaging in complex liver environment, which facilitated sequential catalytic targeted therapy for small HCC. In a carcinogen-induced mouse HCC model, FGP NPs could be used to accurately diagnose HCC in a liver cirrhosis background as well as distinguish HCC nodules from other abnormal liver nodules, such as cirrhosis nodules and necrotic nodules, by dynamic contrast-enhanced photoacoustic imaging. In a mouse xenograft HCC model, highly reactive oxygen species were formed by sequential catalytic reactions, which promoted HCC cell apoptosis, significantly increasing the survival of the model mice. The present study provides a basis for the precise detection and elimination of small HCCs in the complex liver environment.

**Graphical Abstract:**

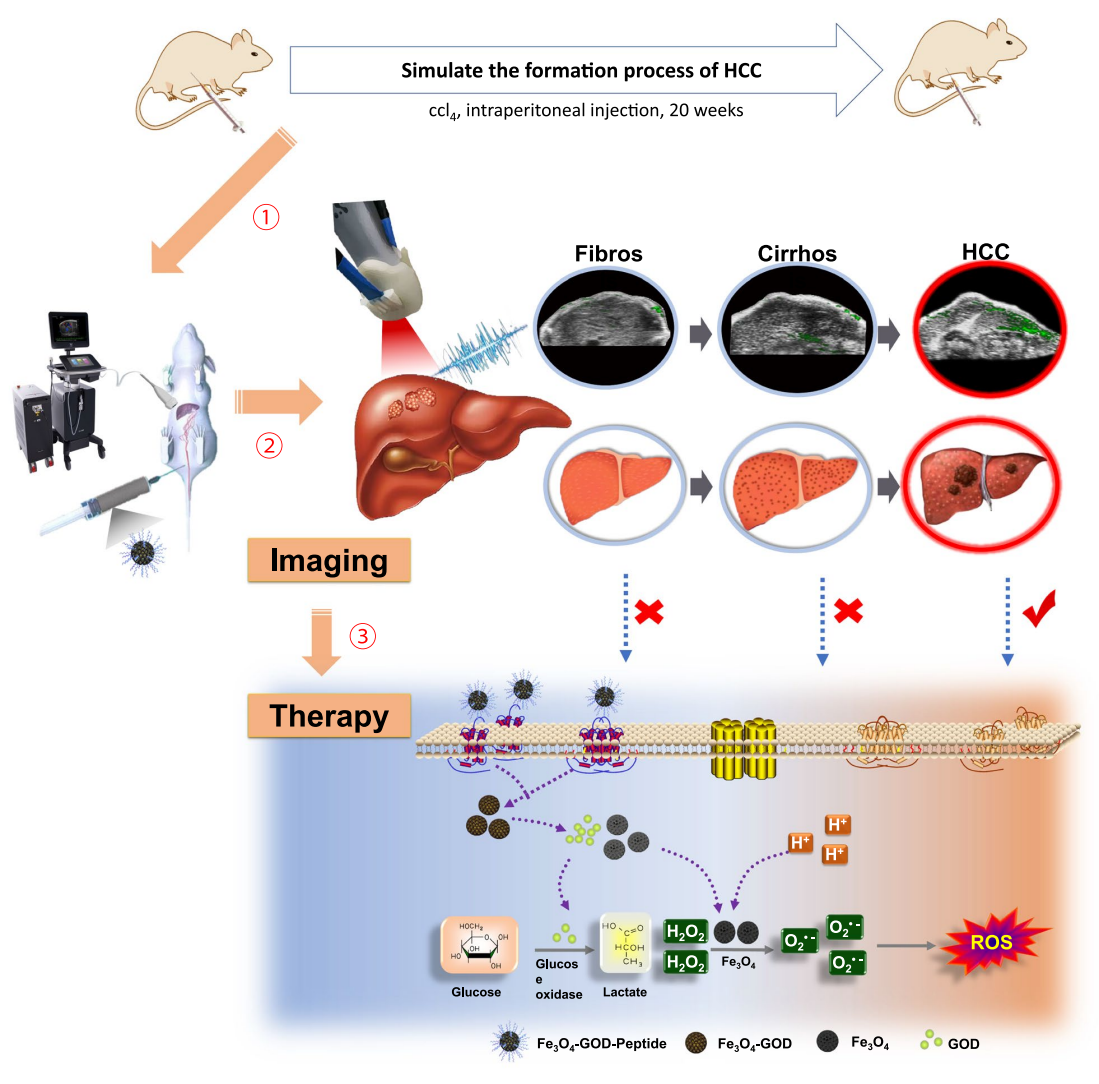

**Supplementary Information:**

The online version contains supplementary material available at 10.1186/s12951-022-01378-w.

## Introduction

Hepatocellular carcinoma (HCC) is the most common histological type of liver cancer, accounting for 90% of liver cancers, with 72% of cases occurring in Asia and more than 50% in China [1, 2]. HCC has a high mortality rate, and most cases are detected at advanced stages when the incidence-to-mortality ratio is 1 [3]. A combination of a lack of early clinical symptoms and the complicated process of liver cancer formation makes it difficult to detect HCC at an early-stage using current diagnostic methods [4]. HCC mostly occurs in patients with chronic liver disease, who may progress from hepatic inflammation, fibrosis, aberrant hepatocyte regeneration, and cirrhosis to HCC [5, 6]. Therefore, HCC typically develops in patients with liver cirrhosis (~ 80% of all HCC cases) [7]. HCC nodules in cirrhotic livers can be classified into regenerative (large regenerative) and dysplastic (low- and high-grade) nodules, early and grade 1 HCC (eHCC-G1), and overt HCC [8]. Owing to intricate process of HCC occurrence and development, clinical guidelines recommend HCC surveillance every 3–4 months in at-risk individuals [9]. Several observational cohort studies comprising patients with cirrhosis or hepatitis B have shown that early-stage HCC patients who undergo cancer monitoring are more likely to receive potentially curative treatments and have improved survival compared with those who receive incidental HCC monitoring [10–12].

Small HCCs are difficult to detect in complex liver environment using currently available imaging methods. The most common surveillance method for detecting early liver nodules is ultrasonography (US), especially for nodules smaller than 1 cm, and US is recommended to be performed at screening intervals of 3–4 months [9]. However, the detection of HCC in the background of cirrhosis by US is a challenge because of the presence of fibrous septa and regenerative nodules, which appear as a rough texture in US images, and these may mask the existence of small tumors [10, 13, 14]. Hence, only a single imaging modality of US does not address the clinical challenges of HCC detection, particularly in cirrhotic livers. Combinations of high spatial resolution equipment [e.g., magnetic resonance imaging (MRI), computed tomography (CT), and photoacoustic imaging (PAI)] could be better methods for detecting early-stage HCC than US. The choice of surveillance equipment must balance high spatial resolution, ease of use, and costs to optimize early HCC detection. Among these methods, CT is less attractive due to its high radiation dose, and MRI due to its high price and long operation time. An innovative technique is urgently required to circumvent these challenges. PAI is an advanced noninvasive imaging technology that can obtain high-resolution anatomical, functional, and molecular imaging in vivo, in which a short laser pulse illuminates the target, and the absorbed light energy is converted into acoustic waves through a series of conversion processes [15]. Combined US/PAI modality, in which US generates structural images and PAI captures tumor signals [16], could enable the diagnosis of small tumors in complex environments. The acoustic signals produced by optical absorption from either endogenous chromophores (e.g., oxygenated or deoxygenated hemoglobin) or exogenous contrast agents, such as organic dyes, gold nanocrystals, and iron oxide noncompounds [17–20], can be used as bioactive molecular tracers in vivo as well as probes for cancer diagnosis and therapy monitoring. Therefore, a targeted HCC PA contrast agent could assist in the accurate diagnosis of small HCCs in complex liver environments.

To detect HCC in a complex liver background accurately, it is necessary to develop a contrast agent with high sensitivity and specificity to target HCC cells. Glypican-3 (GPC3) is a heparan sulfate proteoglycan that functions as a Wnt coreceptor, which is one of the key regulators of HCC tumor progression. It is an oncofetal proteoglycan anchored to the cell membrane and is overexpressed in 70% of HCC tumors but not in healthy adult liver tissue [21]. The progression from precancerous lesions to small liver cancer is usually associated with increased GPC3 expression. GPC3 can distinguish eHCC-G1 from the dysplastic nodules of liver cirrhosis, indicating that GPC3 could be used as a biomarker for the early detection of HCC [8]. Recently, GPC3 has been used as a target for molecular imaging and therapeutic intervention in HCC [22–24]. Our previous study has also confirmed that GPC3-probe can specifically target HCC nodules in the background of normal liver [25].

Once HCC nodules are accurately targeted, an effective treatment strategy is needed to selectively eliminate the cancer cells. Although image-guided therapies using radiation, microwave, and photoacoustic (PA)/photothermal strategies can effectively eliminate cancer cells, these therapeutic methods also damage the normal tissues surrounding the tumor. To achieve targeted and tumor-specific treatment, in addition to targeting GPC3 on HCC cell membranes, the use of Fe_3_O_4_ as a contrast agent for PAI not only enhances the accuracy of small HCC detection but also produces a Fenton-like reaction in the tumor microenvironment (TME). Fe_3_O_4_ can react with H_2_O_2_ to produce water and oxygen at neutral pH, but it can produce toxic reactive oxygen species (ROS) and free hydroxyl radicals (·OH) in the acidic TME [26]. Nonetheless, the amount of ROS in tumor cells is markedly low to achieve sufficient anti-tumor efficacy via this reaction alone. To achieve tumor inhibition, in the present study, a special structural characteristic of the contrast agent was exploited to generate mesoporous Fe_3_O_4_ containing glucose oxidase (GOD). The TME contains high levels of glucose to support rapid tumor growth, and GOD catalyzes glucose into H_2_O_2_, which reacts with Fe_3_O_4_ to produce hydroxyl radicals and kill the tumor cells. ROS are produced via these sequential catalytic reactions, which consume glucose, and thus, the activation of these pathways can starve tumor cells to death [27, 28]. In summary, mesoporous Fe_3_O_4_-containing GOD-conjugated GPC3 peptide nanoparticles (FGP NPs) can bind to the membrane protein GPC3, which is expressed on the surface of HCC cells, and trigger a series of catalytic reactions in the TME.

**Scheme 1:**
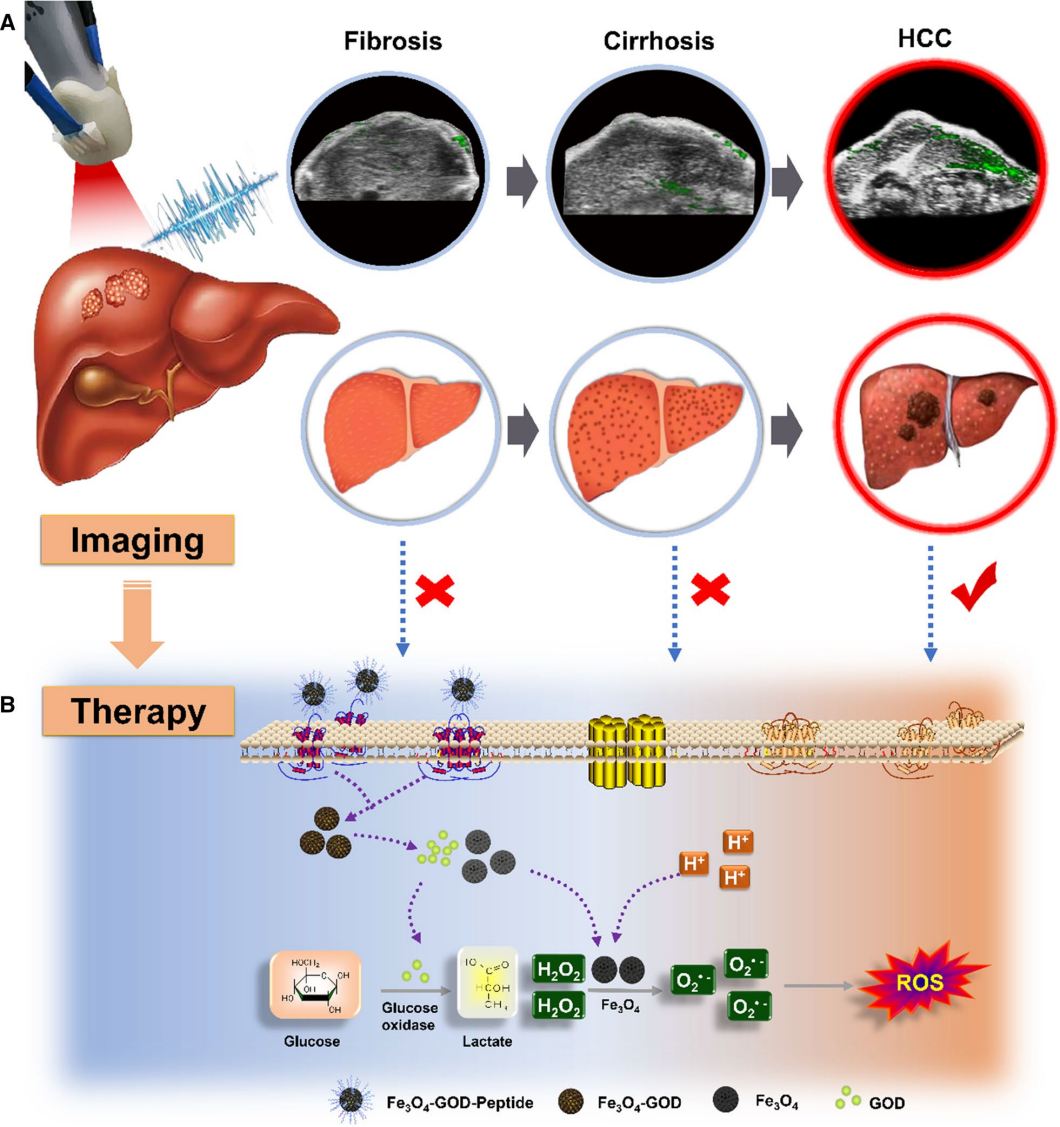
Scheme of targeted detection and sequential treatment of small hepatocellular carcinoma (HCC) in complex liver environment. **A** The development of liver cancer was simulated in vivo using CCl_4_ to induce the formation of liver cancer. Each stage (like fibrosis, cirrhosis and HCC) of this process was imaged by US/PAI after injecting HCC targeted nanoparticles (Fe_3_O_4_-GOD-Peptide, FGP NPs). HCC model had a markedly stronger PAI signal, with low or no signal in fibrosis and cirrhosis model as comparison. **B** After targeting to the GPC3 on the HCC cell membrane, the FGP NPs can only exert the maximum killing effect in the TME because it is mildly acidic and glucose-dependent. Glucose oxidase (GOD) can react with glucose in the tumor cells to produce high levels of H_2_O_2_ and then continue to react with Fe_3_O_4_ to produce toxic ROS-_·_OH radicals in acidic environments, such as extracellular and partial organelles (e.g., lysosomes), to trigger tumor cell apoptosis

In the present study, we aimed to overcome the current lack of effective clinical diagnostic tools for early-stage small HCC in complex liver environment, particularly in cirrhotic or otherwise diseased liver environments. To this end, we applied a targeted detection method (combined US/PAI) as an imaging guide for the application of a sequential catalytic treatment (enzyme and Fenton reaction catalysts (Scheme 1). We determined the critical time point imaging and signal characteristics of HCC formation by PAI to achieve improved diagnosis and identification. Finally, we showed that FGP NPs could specifically and selectively trigger a series of catalytic reactions in the TME to generate ROS, thereby inducing tumor cell apoptosis. This novel strategy could markedly enhance the accuracy of early-stage HCC diagnosis, thus leading to effective early intervention and treatment.

## Results

### GPC3 is specifically expressed on the surface of HCC cells

We first verified that GPC3 is a suitable target for directing HCC detection by immunofluorescence techniques, indicating that this protein is specifically expressed on the surface of HCC cells. To demonstrate specific expression of GPC3 in HCC tissues, we used a common biomarker of HCC arginase (ARG1).

**Fig. 1:**
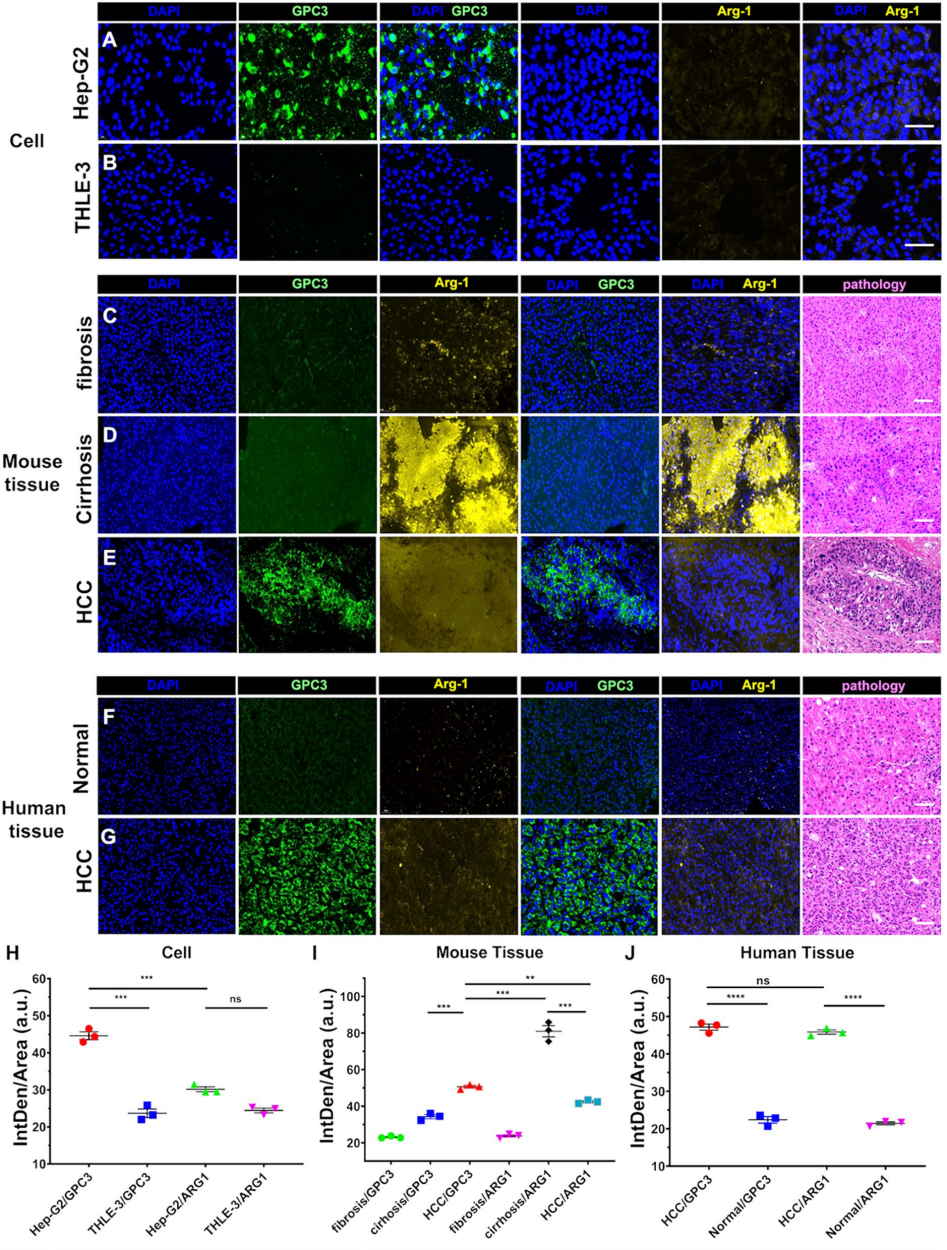
GPC3 is specifically expressed on the surface of HCC cells. **A**, **B** Immunofluorescence staining of the HCC biomarkers, GPC3 (green) and ARG1 (yellow), in Hep-G2 and THLE-3 cell lines. Scale bars = 100 µm. **C**, **D**, **E** Immunofluorescence staining of the HCC biomarkers, GPC3 (green) and ARG1 (yellow), in fibrotic, cirrhotic, and HCC tissues collected from a CCl_4_-treated mouse model of liver cancer. Scale bars = 100 µm. **F**, **G** Immunofluorescence staining of the HCC biomarkers, GPC3 (green) and ARG1 (yellow), in human-derived normal liver and HCC tissues. **H** Quantitative analysis of Immunofluorescence staining of the HCC biomarkers in Hep-G2 and THLE-3 cell lines. **I** Quantitative analysis of Immunofluorescence staining of the HCC biomarkers in fibrotic, cirrhotic, and HCC tissues collected from a CCl4-treated mouse model of liver cancer. **J** Quantitative analysis of immunofluorescence staining of the HCC biomarkers in human-derived normal liver and HCC tissues. Scale bars = 100 µm. *P < 0.05; **P < 0.01; ***P < 0.001, ****P < 0.0001, *ns* not significant

Strong GPC3 fluorescence signal intensity (green) was visible on the surface of human liver cancer Hep-G2 cell membrane and in the intercellular space, but the intensity of ARG1 (yellow) signal was comparatively low (Fig. 1A, B, H). In contrast, in normal liver THLE-3 cells, both ARG1 and GPC3 fluorescence signals were absent. This indicates that GPC3 is expressed in liver cancer cells but not in normal liver cells. However, GPC3 could be more specific for HCC than ARG1, which will be important for differentiating HCC from liver cirrhosis. Therefore, we generated an animal model of liver cancer by injecting mouse with CCl_4_ and then excised tissue samples from the mouse at the three typical stages of HCC progression (liver fibrosis, liver cirrhosis, and HCC) for immunofluorescence staining of GPC3 and ARG1 (Fig. 1C–E). The expression of GPC3 in HCC tissue was higher than that in cirrhosis tissue and in fibrosis tissue (Fig. 1I). The results of this analysis revealed that the GPC3 signal was specific to HCC tissue but not to fibrotic or cirrhotic liver tissue. However, the intensity of ARG1 signal was higher in the cirrhotic liver tissue than in the HCC tissue. Finally, to verify that GPC3 is a highly specific biomarker for liver cancer, we used human tissue samples for verification (Fig. 1F, G). In these samples, both GPC3 and ARG1 were expressed in the liver cancer tissue but not in normal liver tissue, and the expression of GPC3 or ARG1 in HCC tissue were higher than that in normal tissue (Fig. 1J). Therefore, GPC3 is a highly specific biomarker with clinical potential for the diagnosis of liver cancer.

In summary, GPC3 is only expressed in HCC cells, but ARG1 is expressed in cirrhotic liver tissue as well as HCC.

### Characterization of FGP NPs

**Fig. 2:**
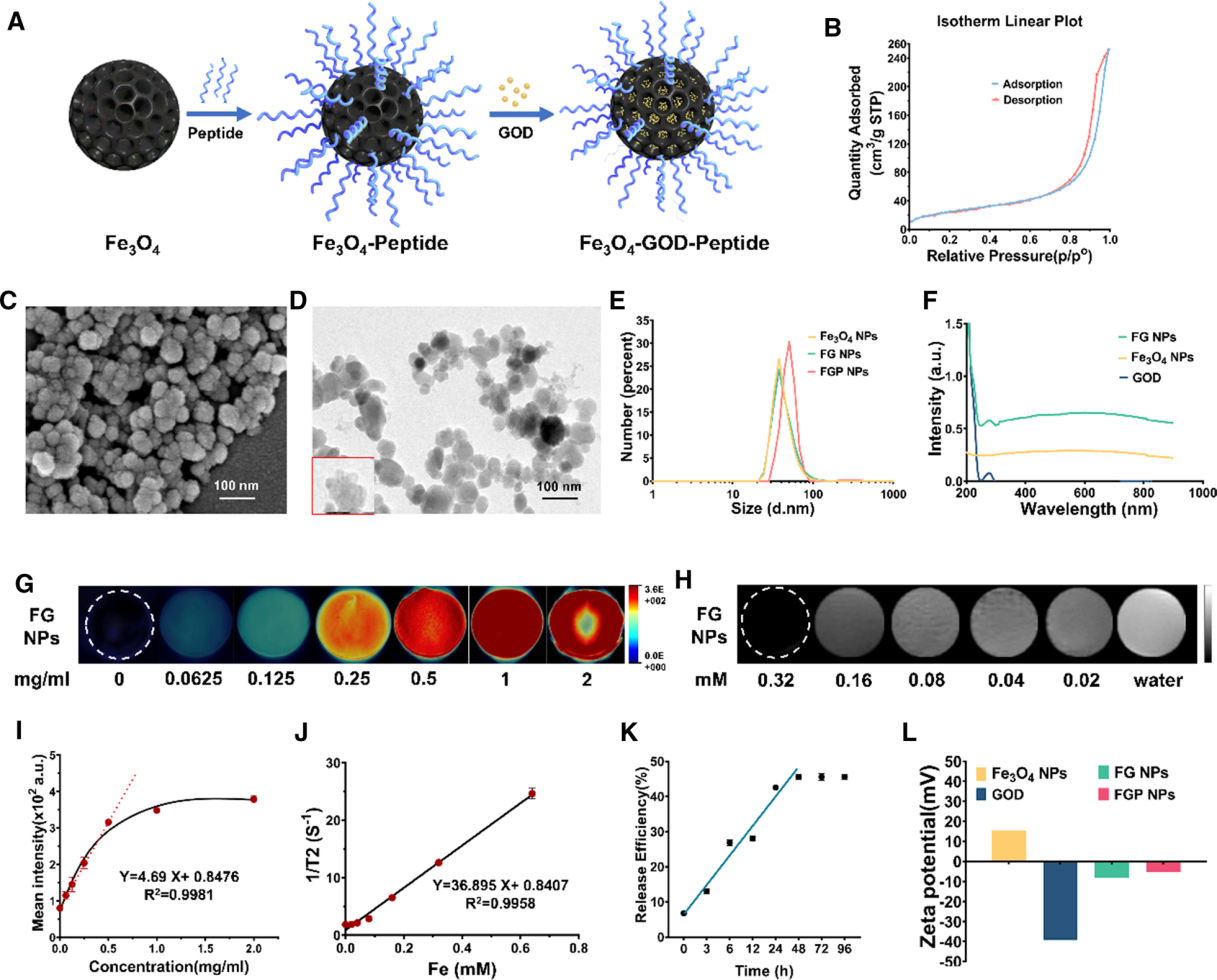
Characterization of the nanoparticle (NP) probe. **A** Schematic of the probe (FGP NPs) fabrication process. **B** Isothermal linear plot of mesoporous Fe_3_O_4_. **C** Scanning electron microscopy (SEM) image of Fe_3_O_4_ NPs. **D** Transmission electron microscopy (TEM) image of Fe_3_O_4_ NPs. **E** Hydrated particle size of Fe_3_O_4_ NPs, Fe_3_O_4_-glucose oxidase (GOD) (FG) NPs, and Fe_3_O_4_-GOD-peptide (FGP) NPs. **F** UV–vis absorption spectra of Fe_3_O_4_ NPs, FG NPs, and GOD. **G** Photoacoustic images and **I** photoacoustic signal of FG NPs in solution at different concentrations. **H** T2-weighted MRI images and **J** T2 relaxation rate of FG NPs in solution at different Fe concentrations. **K** Release rate of GOD from FG NPs over time. **L** The surface potentials of Fe_3_O_4_ NPs, GOD, FG NPs, and FGP NPs

FGP NPs comprise mesoporous Fe_3_O_4_, GOD, and a GPC3-targeting peptide (Fig. 2A). The specific surface area and pore diameter of mesopores Fe_3_O_4_ were confirmed by Brunauer–Emmett–Teller (BET) and Barrett–Joyner–Halenda (BJH) methods, respectively. A BET test revealed that a closed loop formed between the adsorption and desorption curves, thus proving the existence of mesopores (Fig. 2B). The BET surface area was 92.5000 m^2^/g, and the BJH adsorption average pore width (4 V/A) was 15.6342 nm (Additional file 1: Fig. S1A). The size of the pore was sufficient to enable the NPs to contain GOD. Scanning electron microscopy (SEM) images of the mesoporous Fe_3_O_4_ particles revealed a uniform spherical morphology with a cratered structure (Fig. 2C). Since GOD was dispersed in the pores of mesoporous Fe_3_O_4_, the hydrated particle sizes of Fe_3_O_4_ NPs and Fe_3_O_4_-GOD (FG NPs) were both approximately 37.8 nm (Fig. 2E), and the hydrated particle sizes remained stable over 7 days (Additional file 1: Fig. S1B). In contrast, the hydrated size of FGP NPs was slightly large, at approximately 50.7 nm. The diameter of mesoporous Fe_3_O_4_ NPs was approximately 30 nm, which was determined using transmission electron microscopy (TEM) (Fig. 2D). Fourier transform infrared (FT-IR) spectra of Fe_3_O_4_ and Fe_3_O_4_-Peptide was added to further confirm the conjugation of Fe_3_O_4_ with GPC3 peptide. The absorption peak of Fe_3_O_4_-Peptide at 3440 cm^−1^ was lower than that of Fe_3_O_4_ (Additional file 1: Fig. S1C), which is the characteristic peak of N–H. However, at 1650 cm^−1^ [the characteristic peak of C=O (CONH)], the absorption peak of Fe_3_O_4_-Peptide NPs was higher than that of Fe_3_O_4_. These results confirmed that GPC3 peptide was successfully conjugated with Fe_3_O_4_. To demonstrate that GOD was dispersed in mesoporous Fe_3_O_4_ pores, we measured the UV absorption spectra of GOD, Fe_3_O_4_ NPs, and FG NPs. The UV absorption spectrum of Fe_3_O_4_ NPs is a broad-spectrum smooth curve, and the UV absorption spectra of FG NPs is similar to that of GOD which attained peaks at 276 nm (Fig. 2F). The release rate of GOD was determined to fully illustrate the dispersion of GOD in mesoporous Fe_3_O_4_ (Fig. 2K). The initial release rate of GOD from FG NPs was 6.37%, which slowly increased until 48 h, reaching a maximum release rate of 44.79%. This suggested that the maximum amount of GOD dispersed in mesoporous Fe_3_O_4_ could be locally released during the period of probe retained in the tumor in vivo. Subsequently, the surface potentials of Fe_3_O_4_ NPs, GOD, FG NPs, and FGP NPs were measured (Fig. 2L). After modification, the surface potential of FG NPs was − 8.28 mV, and the surface potential of FGP NPs was − 5.21 mV. Finally, the PA and magnetic signal intensities of the FG NPs were measured. PA signal intensity increased with increasing probe concentration (Fig. 2G), and there was a positive correlation (R^2^ = 0.9981) between probe concentration and PA signal intensity (Fig. 2I). When probe concentration reached 0.5 mg/mL, PA signal intensity reached its maximum value of approximately 3 × 10^2^ arbitrary units. FG NPs have an absorption spectrum similar to that of melanin, which absorbs a large amount of light energy and produces a strong acoustic signal. The T2-weighted MRI signal intensity (Fig. 2H) of FG NPs gradually decreased with increasing probe concentration. The relative relaxation rate (R2) of the materials was 36.895 mM^−1^ s^−1^ (Fig. 2J). The linear correlation (R^2^) between probe concentration and T2-weighted imaging signal intensity was 0.9958, suggesting that FG NPs concentration was negatively correlated with the T2-weighted imaging signal intensity.

### Validation of the specificity of FGP NPs for liver cancer

**Fig. 3:**
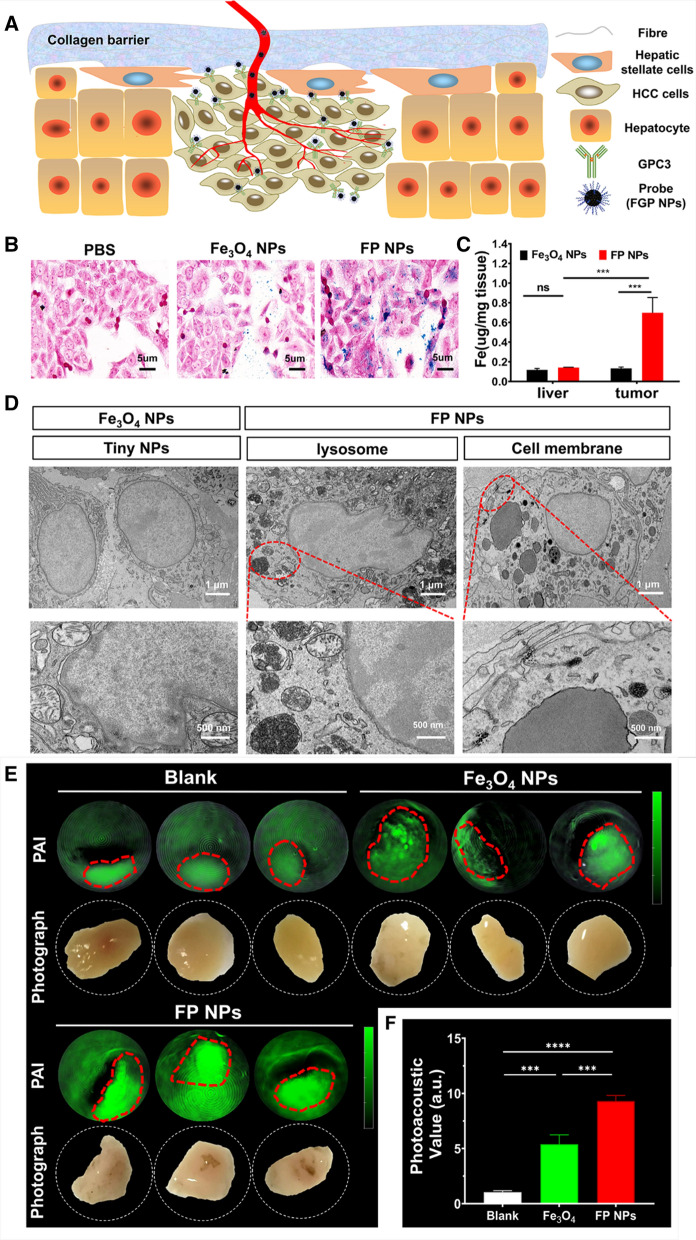
Verification of the targeting specificity of nanoparticles. **A** Illustration of the principle of specific diagnosis of HCC in complex liver environment. **B** Targeting ability of FP NPs. Prussian blue staining after co-incubation of Hep-G2 cells with PBS, Fe_3_O_4_ NPs, or FP NPs. **C** The concentrations of targeted probes (FP NPs) and non-targeted probes (Fe_3_O_4_ NPs) in healthy liver and tumor tissues were measured using ICP-MASS. **D** Targeting ability of FP NPs in vivo. The ultrastructure of HCC cells was obtained by transmission electron microscopy (TEM) with higher magnification (×10,000), and FP NPs were visible in the lysosomes and on the membrane of HCC cells (black aggregation). **E** Photoacoustic imaging of human HCC tissues incubated with saline, Fe_3_O_4_ NPs, or FP NPs. **F** Photoacoustic signal value of different groups (n = 3). ***P < 0.001, ****P < 0.0001; *ns* not significant

Figure 3A illustrates the principle of specific diagnosis of HCC in the context of the complex liver environment. The essential features of a cancer-specific therapeutic agent are as follows: (1) Even in the presence of a collagen barrier, FGP NPs should be able to move into the tumor area with bloodstream. (2) The probe peptides should specifically target tumor cells. (3) FGP NPs should be enriched in the tumor area.

We performed Prussian blue staining (Fig. 3B) to verify that the probe was specifically enriched in the tumor area, which stains the iron element in the HCC cells. Compared with cells in the PBS group, a small number of cells in the Fe_3_O_4_ NP group were stained, but numerous liver cancer cells were stained in the Fe_3_O_4_-peptide nanoparticles (FP NPs) group. We verified this result in vivo (Fig. 3D). The results of TEM analysis revealed the cells receiving a few probes around the cells of the group Fe_3_O_4_ NPs, but a large number of probes are gathered on the cell membrane and in the intracellular lysosomes of cells receiving FP NPs. In the Fe_3_O_4_ NP group, the concentration of probes in the liver was similar to that of probes in the tumor. In the FP NPs group, the concentration of probes was 4.9-fold (p < 0.001) higher in the tumor than in the liver (Fig. 3C). The concentration of Fe_3_O_4_ NPs in the liver tissues of the Fe_3_O_4_ NPs group was similar to that of FP NPs in the liver tissues of the FP NPs group.

At last, human HCC tissues were used to verify the targeting of the probe. The results proved that the photoacoustic signal intensity of the FP NPs group was 8.87-fold (p < 0.0001) and 1.75-fold (p < 0.001) higher than that of the blank group and the Fe_3_O_4_ NPs group, respectively (Fig. 3E, F and Additional file 1: Fig. S2).

### Therapeutic efficacy of the probe in vitro

FGP NPs can trigger a series of catalytic reactions in the TME. GOD reacts with glucose in tumor cells to generate H_2_O_2_, which reacts with Fe_3_O_4_ to produce toxic ROS-·OH radicals that induces apoptosis of cancer cells in acidic TME.

**Fig. 4:**
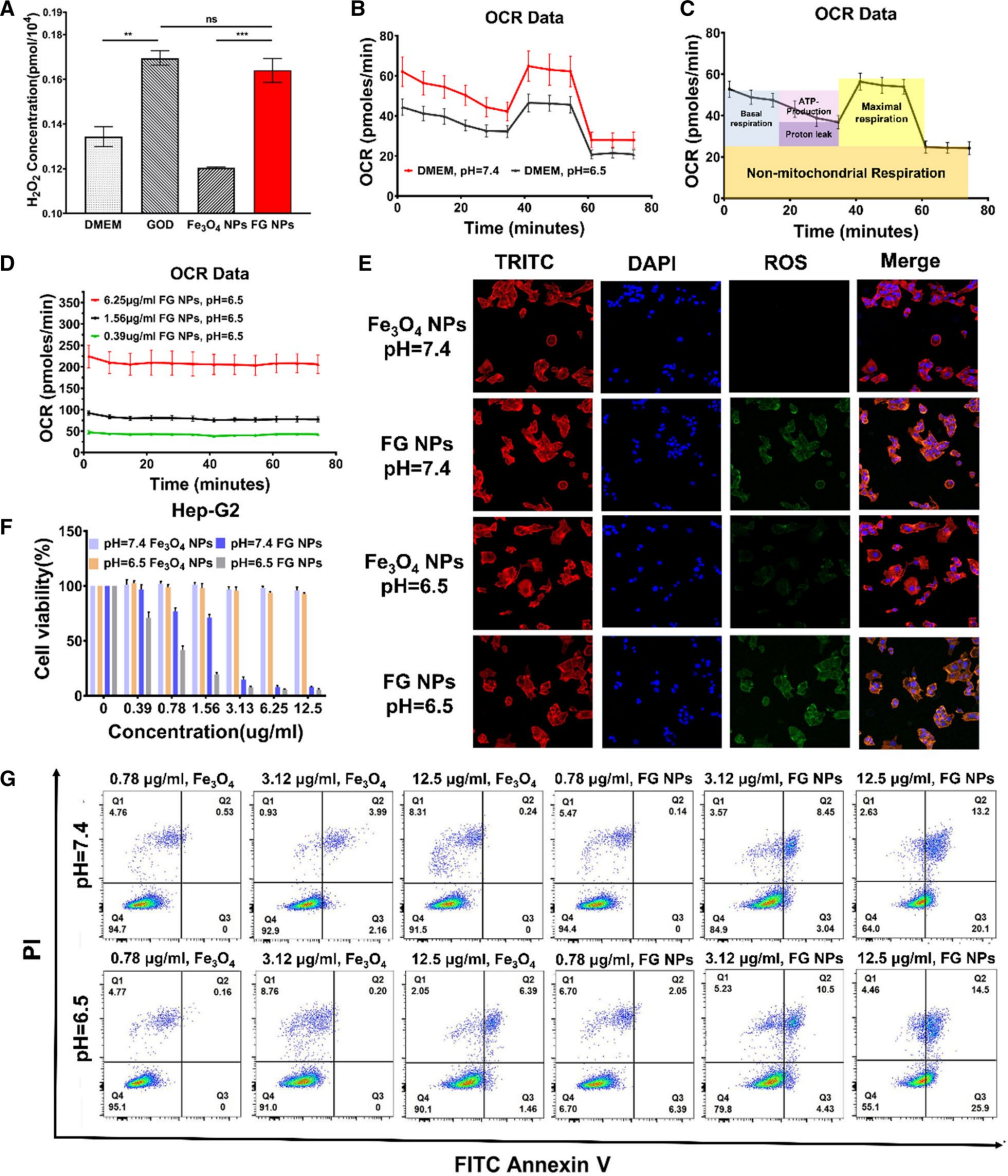
Effect of a targeted anti-cancer probe on human hepatic carcinoma cells. **A** Different probes were used (Dulbecco’s modified Eagle high-glucose medium (DMEM), glucose oxidase (GOD), Fe_3_O_4_ nanoparticles (NPs), Fe_3_O_4_-GOD NPs (FG NPs)) to stimulate tumor cells to produce H_2_O_2_ in neutral (pH = 7.4) media. **B** Average OCR (pmoles/min) of Hep-G2 cells incubate with DMEM (pH = 7.4, n = 3) and DMEM (pH = 6.5, n = 3). **C** Average OCR (pmoles/min) of Hep-G2 cells incubate with 6.25 µg/ml Fe_3_O_4_ NPs (pH = 7.4, n = 3). **D** Average OCR (pmoles/min) of Hep-G2 cells incubate with different concentrations (0.39 μg/ml, 1.56 μg/ml, 6.25 μg/ml) of FG NPs. **E** Fe_3_O_4_ NPs and FG NPs stimulate tumor cells to produce ROS in acidic (pH = 6.5) and neutral media (pH = 7.4). **F** The viability of Hep-G2 cells. **G** Live-dead cell staining kit and flow cytometry analysis of each probe (Fe_3_O_4_ NPs and FG NPs) at different concentrations in acidic (pH = 6.5) and neutral media (pH = 7.4). **P < 0.01; ***P < 0.001, *ns* not significant

In this sequential catalytic process, the production of H_2_O_2_ is essential to the therapeutic efficacy of the nanoparticles. The amount of H_2_O_2_ produced in the culture medium of Hep-G2 cells was more than that produced in the culture medium of cells incubated with Fe_3_O_4_ NPs, which indicates that Fe_3_O_4_ NPs reacted with H_2_O_2_ and depleted it (Fig. 4A). However, the addition of GOD and GOD in the form of FG NPs to the culture medium promoted the production of H_2_O_2_ by HCC cells. H_2_O_2_ concentration in HCC liver cells of the FG NP group was 0.19-fold lower and 2.6-fold (p < 0.05) higher than that of the GOD group and the Dulbecco’s modified Eagle high-glucose medium (DMEM) group, respectively.

We next verified that ROS was produced in the final step of the sequential catalytic reaction (Fig. 4E). The group containing Fe_3_O_4_ NPs alone in culture medium did not generate ROS regardless of the pH of the medium. The group containing FG NPs in culture medium produced more ROS in acidic medium than in neutral medium. Subsequently, a mitochondrial stress test was used to verify the production of ROS in the presence of FG NPs (Fig. 4B–D). Mammalian cells generate ATP via mitochondrial (oxidative phosphorylation) and non-mitochondrial (glycolysis) metabolic pathways. In the Hep-G2 cell line, mitochondria play a key role in energy metabolism and cell cycle regulation. Mitochondrial respiration was evaluated by calculating the maximal oxygen consumption rate (OCR) after adding the electron transport chain inhibitors. The OCR in the Fe_3_O_4_ and blank control groups was similar (Fig. 4B, C), indicating that Fe_3_O_4_ alone or acidic culture medium alone has a minimal effect on the basal ATP production and spare capacity. After adding different concentrations of FG NPs to acidic culture medium, the OCR value increased significantly, and the mitochondria had little spare capacity and ATP production rate (Fig. 4D). Therefore, FG NPs markedly affected the capacity and ATP production rate of mitochondria, during which OCR production significantly increased OCR; ROS production and OCR are positively correlated [29].

Finally, the cytotoxicity of Fe_3_O_4_ NPs was evaluated by testing the effect of different nanoparticles concentrations and pH (Fig. 4F and Additional file 1: Fig. S3A, B). The cell viability was greater than 90% until the Fe_3_O_4_ NPs concentration reached 12.5 μg/ml; this confirmed that Fe_3_O_4_ NPs were non-toxic in vitro. However, cytotoxicity was observed for FG NPs, which was more pronounced in acidic medium than in neutral conditioned medium. At a probe concentration of 1.56 μg/ml, the viability of Hep-G2 HCC cells was approximately 71.26% at pH 7.4 and 18.91% at pH 6.5, the viability trend of LM3 HCC cells was similar to that of Hep-G2 (Additional file 1: Fig. S3A), and the viability of normal liver THLE-3 cells was 94.84% at pH 7.4 and 85.06% at pH 6.5 (Additional file 1: Fig. S3B). Finally, a cell Live Dead Cell staining kit coupled with flow cytometry was used to confirm that FG NPs could kill tumor cells in an acidic environment (Fig. 4G and Additional file 1: Fig. S3C). In summary, while Fe_3_O_4_ itself is not cytotoxic, FG NPs could trigger a series of catalytic reactions to kill tumor cells specifically in the TME.

### Magnetic resonance imaging of carcinogen-induction HCC Mouse model

Since Fe_3_O_4_ NPs show optimal magnetic response, controllable size, suitable biocompatibility and low toxicity, as well as high sensitivity and relaxation rates as T2 contrast agents, they can provide highly accurate information for clinical diagnosis. However, as negative contrast agents, they cannot be used in the diagnosis of micro-tumor tissue.

Additional file 1: Figure S4A shows that FGP NPs can be used as HCC-targeting contrast agents to perform T2 MRI at different stages of liver cancer development. MRI revealed abnormal liver nodules at 14 and 18 weeks after the induction of HCC, but pathology confirmed that the nodules that were visible at 14 weeks were cirrhotic, while the nodules at 18 weeks were micro-HCC nodules (Additional file 1: Fig. S4B). Therefore, FGP NPs in combination with MRI cannot distinguish liver cirrhosis from liver cancer nodules, and thus, it cannot accurately diagnose early-stage HCC.

### The metabolism of the probe in vivo

**Fig. 5:**
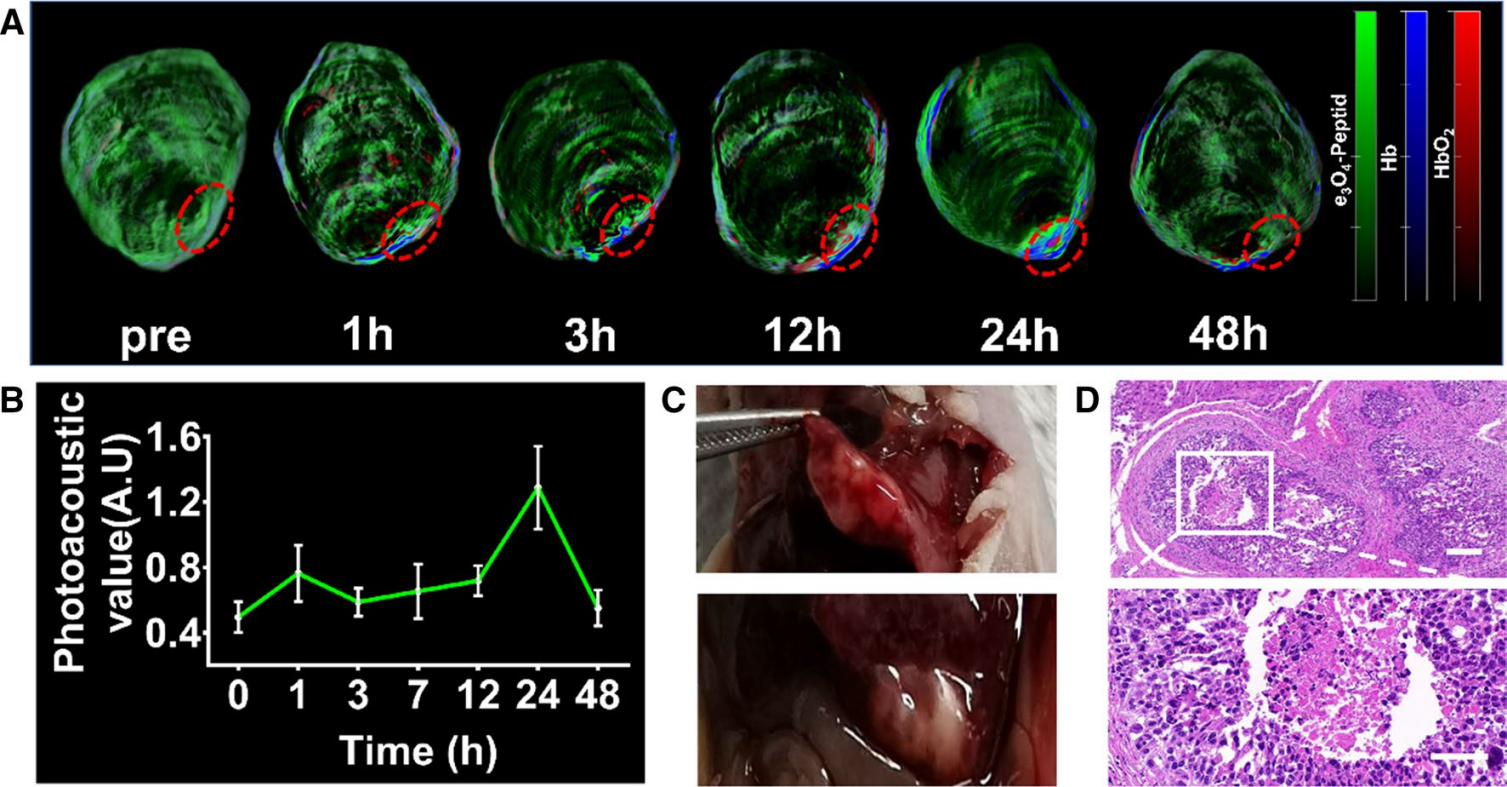
Photoacoustic imaging metabolism of the probe in vivo. **A** Photoacoustic images were obtained before and after the injection of FGP NPs. The tumors were analyzed (**B**) quantitatively, **C** by autopsy, and **D** by histological examination. Scale bar = 50 µm

Although FGP NPs were not effective as MRI contrast agents in the diagnosis of HCC, they can be used as PA contrast agents to assist in the accurate diagnosis of early-stage liver cancer because of their high biological safety, precise targeting and stable PA signal intensity. Xenograft HCC models were diagnosed using PAI, and the metabolism of the probes in the liver was measured (Fig. 5A, B).

The PA signals in the HCC mouse model were analyzed before and at 1, 3, 12, 24, and 48 h after the probe was injected, and the region of interest (ROI) in the tumor was circled for signal intensity analysis (Fig. 5B). The PA signal intensity peaked at 1 h and 24 h, and the signal intensity at 24 h being 1.6-fold higher than that at 1 h. This suggested that the probe bound closely via its targeting peptide to GPC3 on the cancer cell surface and accumulated in large amounts in the tumor. Finally, after acquiring PA images at the 48-h time point, the liver of the mouse was exposed surgically. The actual tumor location was matched to that determined by PAI. Tumors were removed for pathological analysis, the results of which confirmed that the nodules were HCC (Fig. 5D).

### PAI of carcinogen-induction HCC mouse model

We next simulated the development of liver cancer in a mouse model of the disease. This animal model recapitulates the processes of hepatic inflammation, fibrosis, aberrant hepatocyte regeneration, cirrhosis, and finally developing HCC. After injection of targeted probes, cancer development was analyzed by PAI to determine the key points and typical imaging characteristics of HCC during liver cancer development.

**Fig. 6:**
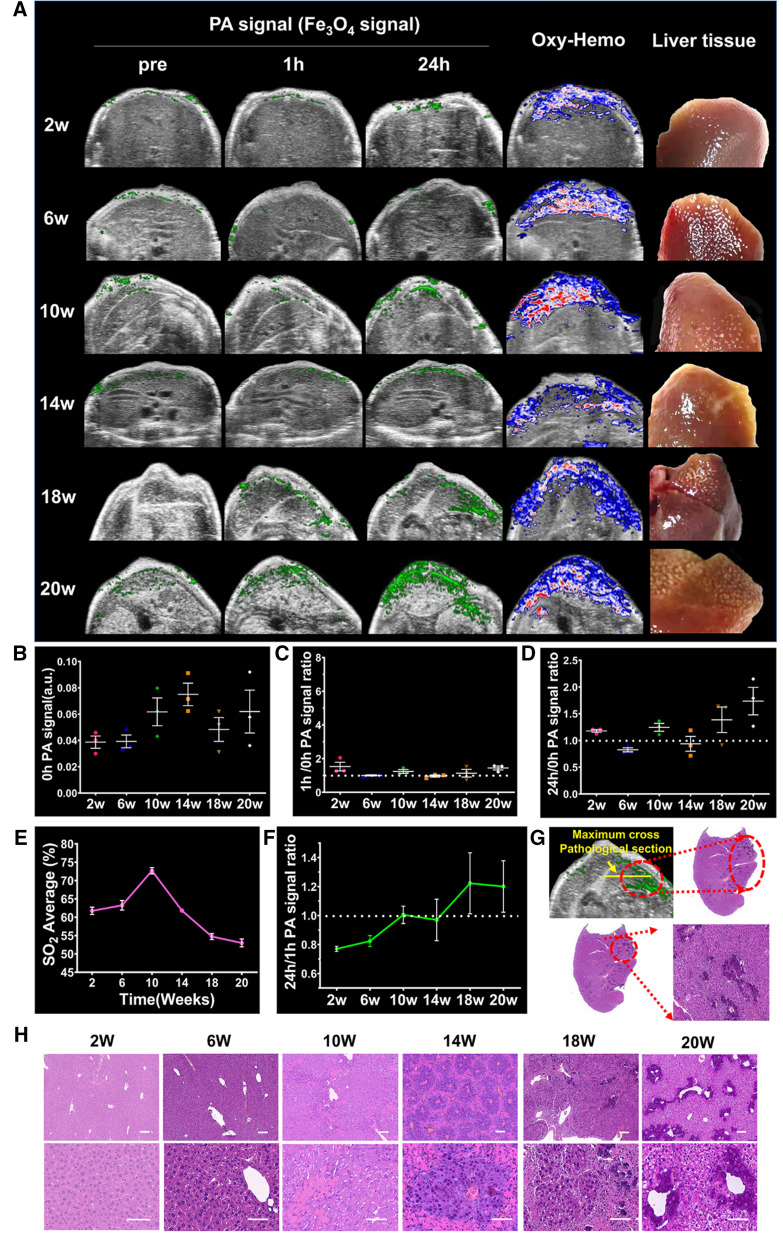
Photoacoustic imaging at different stage following the induction of hepatocellular carcinoma (HCC) in a mouse model. **A** Photoacoustic (PA) images were obtained before and after the injection of FGP NPs at 2, 6, 10, 14, 18, and 20 weeks after HCC induction. Oxygen saturation and autopsy images were acquired at each time point. **B** PA signal intensity before the injection of probe at each time point. **C** The 1:0-h ratio of PA signal intensity at each time point. **D** The 24:0-h ratio of PA signal intensity at each time point. **E** Oxygen saturation at each time point. **F** The 24:1-h ratio of PA signal intensity at each time point. **G** The PA images of the tumor matched the pathology of the dissected organ. **H** Histological examination of liver tissue at different stages of HCC. Scale bar = 50 µm

After imaging, the liver was excised for pathological examination. There were no unique abnormalities in the livers at 2 weeks after HCC induction, but fatty degeneration of hepatocytes and local inflammatory cells were observed at 6 weeks after the induction of HCC. Fibrosis was noticeable at 10 weeks, and typical cirrhosis, in which pseudolobules and tubercles formed, was observed at 14 weeks. Early-stage HCC in the background of liver cirrhosis was observed at 18 weeks, and multiple diffuse liver cancers could be observed at 20 weeks (Fig. 6H). In addition to the appearance of false lobules in the livers, the livers exhibited multi-strip expansion tube structures within the portal vein bloodstream around the main portal vein and branches, which were probably caused by portal hypertension (Additional file 1: Fig. S4C) at 14 weeks after HCC induction. The levels of liver and kidney function markers were within the normal range at 8 weeks, but functional abnormalities occurred from 12 weeks after HCC induction (Additional file 1: Fig. S4D).

Before injecting the contrast agent, we used US to image the progression of HCC. Detection of HCC in the context of cirrhosis is a challenge because of the presence of fibrous septa and regenerative nodules, which appear as a rough texture in US images and may mask the existence of small tumors (Additional file 1: Fig. S5A). PAI of liver cancer stages was performed after PA contrast agent injection (Fig. 6A). The PA signal intensity was similar before injecting the FGP NPs at all cancer stages (Fig. 6B). Subsequently, the retention of the probe in the liver for 1 h was analyzed, and this was represented as the ratio of the 1-h PA signal intensity to the 0-h PA signal intensity. A ratio > 1 indicated that some probes were retained in the liver. The livers at 2 weeks after HCC induction showed the highest 1-h probe retention, and the ratio at the other time points showed a trend toward 1 (Fig. 6C). These phenomena probably occurred because the livers at 2 weeks after disease induction are still “normal”; thus, a large amount of probes can rapidly reach the liver through the blood, while after 6 weeks we observed the appearance of new complex microvessels and fibrous septa, which could reduce the amount of probes that can access the liver. We also analyzed the 24-h retention of the probes in the liver. According to previous experiments, the amount of aggregated probes is the highest at 24 h after injection (Fig. 5B). Only the 24-h retention ratio of the 18- and 20-week post-HCC induction livers were > 1 (Fig. 6D), suggesting that the probe could be retained in the liver for a long period of time because of its close binding with GPC3 on the surface of HCC cells. In contrast, during typical cirrhosis at 14 weeks post-HCC induction, the probe could not be retained for a long time because of the absence of GPC3 expression on the cell surface. To summarize the characteristics of PAI for the diagnosis of HCC, the ratios of the 24-h PA signal intensity to the 1-h PA signal intensity were analyzed (Fig. 6F). In the early stages of HCC development, this ratio was less than 1, indicating that the probe was not retained in the liver at 24-h. In the mid-stages of HCC development (i.e., at 10- and 14-weeks post-HCC induction), this ratio approached 1, indicating that the amount of probes leaving the liver was almost equivalent to the amount entering. Due to the existence of fibrous septum and abnormally tortuous blood vessels, the probe slowly enters and leaves, and easy to be lingering. In the late stages of HCC, (i.e., at 18- and 20-weeks post-HCC induction), this ratio was > 1, suggesting that the probes gradually accumulated in the liver as the probe targeted HCC tissues. This phenomenon was more noticeable in the 3D images (Additional file 1: Fig. S5B). By using this feature, early-stage HCC nodules can be distinguished from other benign liver nodules for accurate identification. According to the criteria summarized above, the 24:1-h ratio of PA signal intensity in livers at 10 weeks post-HCC induction should be lower than that at 14 weeks, but the actual data do not support this hypothesis. This may be related to the maximum blood oxygen saturation at 14 weeks post-HCC induction (Fig. 6E). Blood oxygen saturation is positively correlated with the concentration of probes entering the liver through the bloodstream, and both parameters are positively correlated with the accumulation of probes in the liver [30].

In this study, mouse at 16 weeks after HCC induction had liver nodules smaller than 1 cm in size, and it was impossible to distinguish benign and malignant nodules because the HCC and macroscopic nodules were similar in morphology after dissection. However, data generated using PAI were consistent with this hypothesis. the ratios of the 24-h PA signal intensity to the 1-h PA signal intensity approached 1, indicating that the liver nodule was not HCC nodule (Additional file 1: Fig. S6B). Pathological analysis results confirmed that the small liver nodules were not cancerous but hepatic necrotic nodules (Additional file 1: Fig. S6A). PAI can also accurately identify small HCCs in the background of liver cirrhosis (Fig. 6G). By comparing the dissected tumor and liver with the PA images (the ratios > 1, Additional file 1: S6D), the position where the probes accumulated, as shown in the images, matched the position of the HCC, as determined by pathology (Additional file 1: Fig. S6C). Besides, the accuracy of this dynamic contrast-enhanced PAI method in detecting HCC under complex liver environment was 85.7% (12 out of 14 samples were evaluated correctly, Additional file 1: Fig. S7).

### Sequential catalysis-targeted therapy in vivo

Carcinogen-induced models of in vivo therapies are difficult to standardize, which results in a complicated experimental design. To test the therapeutic efficacy of FGP NPs, a xenograft orthotopic liver cancer model was established [31].

**Fig. 7:**
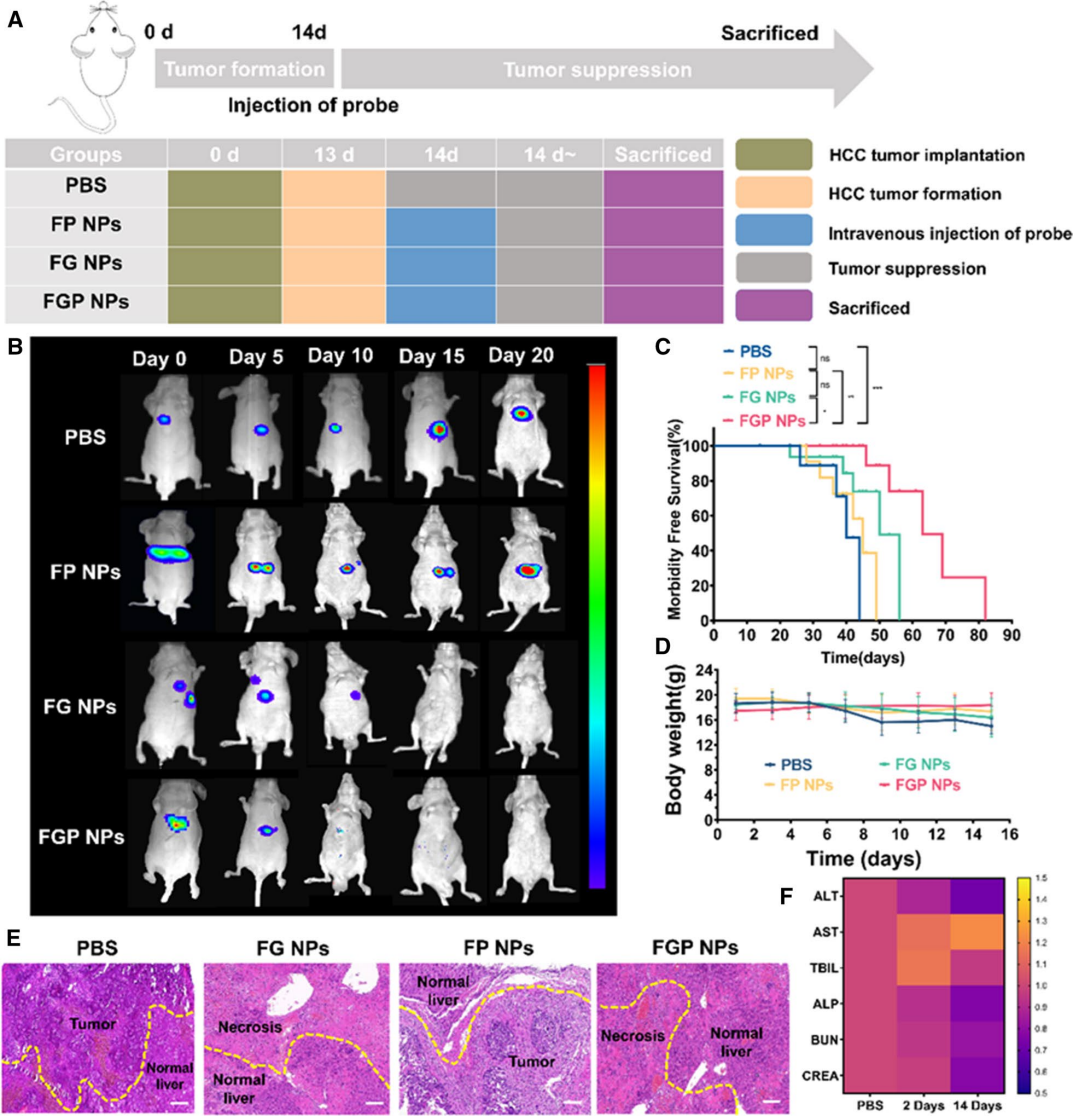
Treatment effects of different nanoparticles. **A** Treatment schedule of xenograft orthotopic liver cancer model mouse. **B** HCC bioluminescence images were under surveillance in each treatment group. **C** Survival after treatment in each group. **D** The body weight changes observed in xenograft orthotopic liver cancer model mouse in each treatment group. **E** H&E staining of tumor tissue. Scale bar = 50 µm. **F** Heat map of liver function and kidney function indexes. *P < 0.05; **P < 0.01; ***P < 0.001, *ns* not significant. PBS: control, FP NPs: Fe_3_O_4_-peptide nanoparticles, FG NPs: Fe_3_O_4_-glucose oxidase (GOD) NPs, FGP NPs: Fe_3_O_4_-GOD-peptide NPs

Mouse bearing Hep-G2 tumors were randomly divided into four groups (n = 5 per group), when the tumor autofluorescence signal intensity reached approximately 1 × 10^4^, these four groups were treated differently and divided into: blank control (PBS), FP NP, FG NP, and FGP NP groups (Fig. 7A). After receiving different treatments, the autofluorescence signal intensity of the tumors in the FP group increased continuously and became similar to that of the tumors in the PBS group. The autofluorescence signal intensity of the tumors in the FGP and FG groups gradually decreased, and the former decreased faster than the latter (Fig. 7B). This confirmed that the Fe_3_O_4_-GOD component of FGP NPs exerts a therapeutic effect on small HCCs, and the peptide portion enhances this therapeutic effect. We also monitored the survival, weight, and pathology of xenograft mouse in each group. FGP NPs significantly prolonged survival with statistical significance (Fig. 7C). The body weight did not change significantly between the groups for the entire duration of the experiment (Fig. 7D). The PBS and FP groups had diffuse tumors, while the FG and FGP groups had several necroses in addition to normal liver tissue (Fig. 7E). Finally, it was confirmed that FGP NPs had no long-term toxicity by detecting liver function, kidney function, and multiple organ pathology were similar in mouse at 2 days and 2 weeks after treatment with nanoparticles (Fig. 7F and Additional file 1: Fig. S8A). In addition, treatment with FGP NPs increased glucose consumption (Additional file 1: Fig. S8B). Taken together, these results indicate that FGP NPs can promote tumor cell apoptosis, thereby selectively eliminating tumor tissue.

## Conclusion

Hepatocarcinogenesis is a multistep and complex process. Small liver nodules may also be regenerative or dysplasia nodules, which are the precursors of HCC, and most HCCs appear in patients with cirrhosis. Therefore, the accurate diagnosis of early-stage small HCC in the complex liver environment remains challenging. The imaging methods commonly used to clinically diagnose HCC include CT, MRI, and US. Only 68% of HCC tumors with a diameter of 1–2 cm were detected by contrast-enhanced CT [32]. Furthermore, because of its radioactivity, it cannot be used as an imaging method for the early screening of liver cancer. Although MRI has high spatial resolution to detect small HCC nodules, the identification of nodules with diameters less than 1 cm in the background of cirrhosis remains challenging [33]. For its high price and long scanning time, MRI cannot be used as an imaging method for the early screening of liver cancer. Surveillance by US at early-stage of HCC is recommended because it is simple to use, efficient, and cost-effective. However, as the first step of the diagnosis for detection of HCC in cirrhosis, the sensitivity of US is only 45%, resulting in only 4 of 10 HCCs being detected at early-stage [34]. Therefore, there is an urgent need to develop a new instrument or method for combined use with US for screening early HCC in the complex liver environment. In the present study, we tested an advanced non-invasive imaging technology combining PAI and US for high-resolution anatomical, functional, and molecular imaging, which can be used for the accurate detection of small HCCs. PA has a superior resolution (down to 30 µm) and deeper imaging capabilities (up to 4 cm) [35]. Based on the structural images provided by US, high-resolution PA combined with highly specific probes, such as FGP NPs, can accurately identify HCC signals.

Dynamic contrast-enhanced PAI (DCE PAI) has been applied to analyze the characteristics of small HCC signals. DCE PAI is a novel non-invasive imaging modality that provides a model with high temporal and spatial resolution. Nie et al. obtained the dynamic processes of the Indocyanine Green (ICG) curve in liver fibrosis to understand the PA functional parameters relative to the development of fibrosis, such as the maximum peak time and half-life of ICG [36]. In the present study, the dynamic contrast-enhanced PAI of FGP NP curves were acquired in the process of tumor formation to get HCC typical imaging characteristics that distinguish it from other liver nodules.

Tumor cell metabolism differs from that of normal cells. The TME has certain notable characteristics that can be used for designing diverse tumor-specific therapeutic strategies. Recently, sequential catalytic nanomedicine has attracted considerable research attention, as diversified, highly selective, and highly specific catalytic reactions to achieve the selective killing of tumor cells with minimal side effects on normal tissue. Gu et al. developed a core–shell-based “nanodepot,” consisting of a liposomal core and crosslinked gel shell, to induce cell death by the action of sequential particles that target different parts of cancer cells with two drugs [37]. Shi et al. developed mesoporous silica nanoparticles (NPs) as a carrier to transport iron gall (GA-Fe) to cancer lesions, which augmented the efficacy and safety of cancer therapy [38]. In the present study, based on FGP NPs enriched in the tumor tissue under the guidance of cancer cell-specific targeting peptides, we used the characteristics of the TME to design a sequential catalytic nanoparticle (NP) to trigger tumor cell apoptosis without harming normal cells. However, sequential catalytic treatment is only suitable for small HCCs, and its effect is weak in large HCCs and metastatic HCCs.

In summary, our results indicated that combined US/PAI could detect small HCCs in complex liver environments as well as be used as an imaging guide for targeting sequential catalytic treatment for small HCCs.

## Materials and methods

### Synthesis of FGP NPs

Nanoparticles (NPs) consisting of mesoporous Fe_3_O_4_, GOD, and peptides were generated. Mesoporous Fe_3_O_4_ was synthesized in an oxygen-free environment, with the entire reaction process being carried out under nitrogen. Iron chloride hexahydrate (10.032 g, Acros) and ethylene glycol (3 ml, Aladdin) were vigorously stirred for 30 min in a conical flask. After obtaining a clear yellow solution, anhydrous sodium acetate (3.0017 g, Acros) and diethylenetriamine (3.8 ml, Acros) were added. The solution transferred to reaction still, and conditions of which were set to 180 °C for 4 h. After the reaction was completed, the mixed solution was cooled to room temperature, washed three times with ultrapure water, and dried in a vacuum drying box at 50 °C. Subsequently, 10 mg of the synthesized Fe_3_O_4_-NH_2_ was dissolved in 1 ml ethanol ultrasonically. Next, 300 μl of glutaraldehyde solution (25 wt%) was added to the mixture and stirred at room temperature for 6 h. After the sediment was washed three times, a peptide solution (0.2 mg/ml) was mixed with Fe_3_O_4_ and incubated at 4 °C for 6 h. Then, 10 μl of bovine serum albumin (10 wt%) was added, and the mixture was incubated at 4 °C for 2 h. Thereafter, sodium borohydride solution (50 μl, 25 mg/ml) was added to the mixture and incubated at 4 °C for 1 h. The peptide used was a 12-mer peptide (sequence: DHLASLWWGTEL) [39] corresponding to human GPC3. The resulting Fe_3_O_4_-peptide NPs were separated by centrifugation. To generate FGP NPs, 5 mg of FP NPs and 1.6 mg of GOD were ground thoroughly and then dried for 24 h in a vacuum freeze dryer [40, 41].

### Characterization

The hydrodynamic diameters and zeta potential of Fe_3_O_4_ NPs, FP NPs, and FGP NPs were measured using Zetasizer Nano (ZS) (Malvern Instruments, UK). The nitrogen adsorption–desorption isothermal curve and corresponding pore size distribution of Fe_3_O_4_ were determined using a Micromeritics ASAP 2020 PLUS HD88 instrument. TEM (JEM-F200, JAPAN) and SEM (Hitachi S4800, JAPAN) were used to acquire the morphological images of the particles. The optical absorbance spectra were measured using a UV-3600Plus spectrophotometer (SHIMADZU, JAPAN). Fourier near-infrared spectroscopy were determined using a Nicolet iS50 FTIR spectrometer (Thermo Scientific, US). A BCA Protein Assay Kit was used to measure the relaxation rate of GOD in the mesopores of FG NPs. Different concentrations of FG NPs were scanned using a 1.5-T MRI scanner to obtain T2-weighted images, and the relaxation rates were calculated. Using agar as a carrier, the PA signals of FG NPs at different concentrations were measured using multispectral optoacoustic tomography (iTheraMedical, Germany).

### In vitro experiments

Human HCC cell lines (Hep-G2 and LM3) and a normal liver cell line (THLE-3) were obtained from the Cell Resource Center (IBMS) and cultured in DMEM (Gibco) supplemented with 10% fetal bovine serum (Gibco) and 1% penicillin/streptomycin at 37 °C in an atmosphere containing 5% CO_2_.

To measure immunofluorescence in cells, mouse tissue, and human tissue, immunoblotting was performed as previously described [42]. GPC3 expression on the surface of liver cancer cells was evaluated using anti-GPC3 (ab129381) antibodies purchased from Abcam (Cambridge, UK) and anti-ARG1 (mAb #93668) procured from Cell Signaling Technology (USA).

To measure in vitro cytotoxicity, Hep-G2, LM3, and THLE-3 cell lines were tested using the CCK8 Assay Kit. Cells (1 × 10^4^) were seeded onto 96-well plates and incubated for 24 h. Test compounds were added to each well and incubated for 24 h at 37 °C in an atmosphere containing 5% CO_2_. The following test compounds were used: (1) Fe_3_O_4_-peptide NPs at concentrations of 0.39, 0.78, 1.56, 3.125, 6.25, and 12.5 µg/ml in DMEM (pH = 7.4); (2) Fe_3_O_4_-peptide NPs at the same concentrations in DMEM (pH = 6.5); (3) FGP NPs at the same concentrations in DMEM (pH = 7.4); and (4) FGP NPs at the same concentrations in DMEM (pH = 6.5). Cytotoxicity was evaluated using the Cell Counting Kit-8 assay (Solarbio) according to the manufacturer’s instructions.

To determine H_2_O_2_ content in vitro, Hep-G2 cancer cells were seeded onto two 6-well plates at a density of 1 × 10^6^ cells per well for 24 h. The culture medium was replaced by DMEM (pH = 7.4), GOD (pH = 7.4, 6.25 µg/ml), Fe_3_O_4_ (pH = 7.4, 6.25 µg/ml), FG NPs (pH = 7.4, 6.25 µg/ml). After incubation for 4 h, the cells were collected in a centrifuge tube, the supernatant was discarded, and the H_2_O_2_ content was measured using a Micro H_2_O_2_ Assay Kit (Solarbio) according to the manufacturer’s instructions.

To determine ROS content in vitro, Hep-G2 cells were seeded onto confocal dishes at a density of 1 × 10^6^ cells per well and incubated with Fe_3_O_4_ (pH = 7.4, 6.25 µg/ml), Fe_3_O_4_ (pH = 6.5, 6.25 µg/ml), FG NPs (pH = 7.4, 6.25 µg/ml), or FG NPs (pH = 6.5, 6.25 µg/ml) for 4 h. DCFH-DA (emission wavelength of 488 nm) was used to stain ROS inside the cells, and DAPI (emission wavelength of 360–400 nm) was used to stain the nucleus. The distribution of intracellular F-actin was visualized by staining fixed intact cells with TRITC phalloidin (emission wavelength of 540–546 nm). Images were captured using a confocal microscope (Zeiss LSM780).

For the mitochondrial stress test, 1 × 10^4^ cells were seeded onto Seahorse XFp cell culture miniplates using the appropriate cell culture growth medium. After culturing for 24 h, probes at different pH and concentrations were added to the culture medium and incubated for 1 h. The culture medium was replaced with XF medium (pH 7.4) and XF supplements, and the cell culture microplates were incubated at 37 °C without CO_2_ for 45 min at 1 h prior to the assay. Mitochondrial stress was determined using the Seahorse XF Cell Mito Stress Test Kit, according to the manufacturer’s instructions. Open pouch and remove the three tubes containing oligomycin, FCCP, and rotenone/antimycin A. Resuspend content in each tube with the prepared assay medium in volumes. Using a pipette, the medium was gently pipetted up and down the medium (~ 10 times) to solubilize the compounds. These three compounds were added in a certain volume to the wells of the culture plate. Finally, putting which into the instrument (Agilent Seahorse XF, USA) for the mitochondrial stress test.

Apoptosis rates were determined using a calcein-AM/PI double stain kit and flow cytometry. Hep-G2 cells were seeded onto confocal dishes at a density of 1 × 10^6^ cells per well and incubated with Fe_3_O_4_ (pH = 7.4, 6.25 µg/ml), Fe_3_O_4_ (pH = 6.5, 6.25 µg/ml), FG NPs (pH = 7.4, 6.25 µg/ml), or FG NPs (pH = 6.5, 6.25 µg/ml) for 4 h. DCFH-DA (emission wavelength of 488 nm) was used to stain ROS inside the cells, and DAPI (emission wavelength of 360–400 nm) was used to stain the nucleus. The distribution of intracellular F-actin was visualized by staining fixed intact cells with TRITC phalloidin (emission wavelength of 540–546 nm). Images were captured using a confocal microscope (Zeiss LSM780, Germany).

GPC3 is an oncofetal proteoglycan that is anchored to the HCC cell membrane. A Prussian blue assay was used demonstrate GPC3 targeting in vitro. Hep-G2 cells were seeded onto 6-well plates at a density of 1 × 10^6^ cells per well and incubated with DMEM, Fe_3_O_4_ (6.25 µg/ml), or FP NPs (6.25 µg/ml) for 24 h. After the cells were fixed with 4% paraformaldehyde for 15 min, Perls stain was used to bond the internal and external iron contents of the cells prior to staining.

### Animal experiments

To establish an orthotropic liver cancer mouse model, two different methods were applied: xenografting and carcinogen-induction. All mouse were purchased from Beijing Vital River Laboratory Animal Technology Co., Ltd., and raised in the Animal Research Center of the Beijing Key Laboratory of Molecular Imaging of Chinese Academy of Sciences under the condition of constant temperature, constant humidity and free access eating and drinking. To generate the xenograft model, BALB/c athymic nude mouse (5-week-old, male, body weight: 14 to 16 g) were anesthetized with phenobarbital sodium. The mouse was fixed in a supine position, and the layers of skin and peritoneum were cut at approximately 0.8 cm below the xiphoid line at the ventral midline, followed by implantation of Cell suspensions containing 1 × 10^6^ Fluc Hep-G2 cells and Matrigel at a 1:1 (v/v) ratio into the left lobe of the liver. The peritoneum and skin were sutured using 7-0 silk. To assess tumor growth, a small animal optical molecular imaging system (IVIS Imaging Spectrum System, PerkinElmer, USA) was used after 14 days. The bioluminescent signal of orthotropic xenograft liver cancer was generated by the interaction of luciferase from Hep-G2 cells with d-luciferin solution (15 mg/ml, PerkinElmer), which was injected into the abdomen before imaging. To generate the carcinogen-induced HCC model, a CCl_4_ (2.5 μl/g) solution, which was diluted in olive oil to a concentration of 30% v/v, was injected intraperitoneally into BALB/c white mouse (5-week-old, male), 2 times a week for 20 weeks until HCC development. The carcinogen-induced HCC models were confirmed by biopsy every 4 weeks.

To confirm the targeting ability of FP NPs in vivo, TEM of biological tissue was also performed. Fe_3_O_4_ (0.8 mg/ml) or FP NPs (0.8 mg/ml) were injected into HCC mouse (n = 3) through the tail vein. After 24 h, the mouse were perfused with PBS and 2.5% glutaraldehyde, following which the mouse were dissected and 1 × 1 cm liver cancer tissue slices were obtained and soaked in 2.5% glutaraldehyde for fixation. Finally, the distribution of the probes in the cells was imaged using tissue electron microscopy (FEI Tecnai F20). ICP-MS was also used to determine the concentration of Fe_3_O_4_ and FP NPs in vivo. Fe_3_O_4_ (0.8 mg/ml) or FP NPs (0.8 mg/ml) were injected into HCC mouse (n = 3) through the tail vein. After 24 h, the liver and tumor tissues were removed and stored at − 80 °C. Organs of the same weight (approximately 100 mg) were finely ground. The ground tissue was digested with nitric acid (HNO_3_) and 20% hydrogen peroxide (H_2_O_2_) at 37 °C for 24 h. Subsequently, the sample was diluted with 2% nitric acid and filtered through a 0.22-μm membrane. Finally, the concentration of Fe in the samples was quantitatively analyzed by ICP-MS (Agilent ICPMS7800) to evaluate the distribution of the NPs.

For PAI in vivo, HCC model establishment (n = 3) was confirmed using IVIS after 2 weeks of xenograft orthotopic liver cancer development. FP NPs were intravenously injected, and HCC model mouse were placed in the Imaging chamber after anesthetization with phenobarbital sodium. PAI (MSOT; iTheraMedical, Germany) of the complete hepatic region before and after injection was performed at different time points (1, 3, 7, 12, 24, and 48 h), and the PA signal intensities of the tumors and major organs were quantitatively analyzed using MSOT. To further confirm the formation of tumors, liver tissues suspected of containing tumors were removed for pathological examination.

For MRI of the progression of liver cancer, a carcinogen-induced mouse model was used. To generate the carcinogen-induced mouse model, BALB/c athymic white mouse (4-week-old, male, body weight: 14 to 16 g) got Intraperitoneal injection with CCl_4_ (2.5 μl/g) twice a week Magnetic resonance images of the livers of mouse mice at different time points (2, 6, 10, 14, 18, and 20 weeks) (n = 3) after treatment with CCl_4_ (2.5 μl/g) were obtained. Prior to imaging, FP NPs were intravenously injected. HCC model mouses were placed in the imaging housing after anesthetization with phenobarbital sodium, and MRI of the complete hepatic region was performed before and after injection at different time points (1 and 24 h). The representative imaging parameters of the T2-Weighted images were as follows: repetition time (TR) = 3720 ms, echo time (TE) = 66.352 ms, slice thickness = 1 mm. Finally, the liver tissue was removed for pathological examination.

PAI (Visual Sonic 3100 + LAZR, Germany) of the progression of liver cancer was performed in carcinogen-induced model mouse. PAI of livers was conducted at different time points (2, 6, 10, 14, 18, and 20 weeks) (n = 3) after intraperitoneal injection with CCl_4_ (2.5 μl/g) to visualize the progression of liver cancer. HCC model mouses were placed in imaging chamber after anesthetization with phenobarbital sodium, following which PAI of the complete hepatic region was performed before and after FP NP injection at different time points (1 and 24 h). The ROI was determined, and the PA signal intensity and oxygen saturation of the ROI was quantitatively analyzed using Visual Sonic software. Finally, remove 4 pieces of liver tissue from each mouse for pathology, and then compare the pathological results with the photoacoustic imaging results.

To determine the therapeutic efficiency of FGP NPs, HCC model establishment was confirmed using IVIS when the tumor autofluorescence signal intensity reached approximately 1 × 10^4^ after 2 weeks of xenograft orthotopic liver cancer development. We randomly divided mouse into four groups (n = 5), and each group was injected with PBS, FG NPs, FP NPs, or FGP NPs through the tail vein. The treatment effect on each group was determined by measuring the body weight, survival, and tumor signal intensity, which was quantitatively analyzed by measuring the bioluminescent signal intensity of orthotropic xenograft liver cancer using IVIS. Finally, the liver tissue was excised for pathological examination.

### Human tissue experiments

The use of human tissues in the experiments have been approved by the Ethics Committee of Zhujiang Hospital of Southern Medical University (2018-GDYK-002). A surgical specimen of human HCC border tissue with a thickness of about 3 mm was cut out. After the specimen was isolated, it was soaked and stained with different solution (saline, Fe_3_O_4_ NPs and FP NPs) for 2 h. The specimen was washed with saline three times. Then, the specimen was imaged by photoacoustic imaging. At last, the specimens were subjected to HE staining pathological examination, and then compared with the photoacoustic imaging results for verification.

### Statistical analysis

Experimental data were analyzed using GraphPad Prism 9 or SPSS software. All data are expressed as the mean ± SEM. Statistical differences between groups were analyzed using independent two-sample t-tests. The Kaplan–Meier method was used to analyze survival curves, and the log-rank test was used to analyze the statistical differences between survival curves. Differences between groups were considered significant at P < 0.05.

## Supplementary Information

**Additional file 1: Figure S1.** Characterization of probe. (A) BJH desorption Dv/DW pore volume of mesoporous Fe_3_O_4_ NPs. (B) stability of Fe_3_O_4_ NPs (0.4 mg/ml) measured for 7 days. Inset: A photo of Fe_3_O_4_ NPs in various solutions (from left to right: water, saline, DMEM cell medium and serum). (C) Fourier transform infrared spectra of Fe_3_O_4_ and Fe_3_O_4_-Peptide. **Figure S2.** Pathology of Human HCC tissues. H&E staining sections of different human HCC tissue groups (blank, Fe_3_O_4_ NPs, FP NPs). Blank: saline, FP NPs: Fe_3_O_4_-peptide nanoparticles. **Figure S3.** probe in human hepatic carcinoma cells. (A) Cell viability of LM3. (B) Cell viability of THLE-3. (C) Fluorescence microscope images of Calcein AM/PI co-stained with different probes (Fe_3_O_4_ NPs, FG NPs) at different concentration in acidic environment (pH = 6.5) and neutral environment (pH = 7.4). FG NPs: Fe_3_O_4_-GOD nanoparticles. **Figure S4.** MRI metabolism of the probe in Carcinogen-induced mice models. (A) Comparison of the liver tissues before and at 24 h after injection of FGP NPs at different time points (2, 6, 10, 14, 18, 20 weeks) during liver cancer development. Red-dotted circles represent liver nodules. (B) H&E staining sections of liver nodules of 14-week induced mice and 18-week induced mice detected by MRI. (C) In the contrast of 2-week induced mice ultrasound, 14-week induced mice with liver cirrhosis showed obvious portal vein expansion on ultrasound. And the autopsy of which showed obvious portal vein expansion. (D) Liver function and kidney function of normal mice (control), 8-week induced mice and 12-week induced mice. **Figure S5.** Ultrasound and 3D-Photoacoustic imaging of the process of inducing liver cancer formation. (A) Ultrasound of at different timepoints (2w, 6w, 10w, 14w, 18w, 20w) of the process of inducing liver cancer formation. (B) 3D-Photoacoustic imaging at different stages (2w, 6w, 10w, 14w, 18w, 20w) of the process of inducing liver cancer formation. **Figure S6.** Photoacoustic imaging and its signal value of necrosis nodule, HCC nodule and cirrhosis nodule. (A) Autopsy liver necrosis nodules and their corresponding pathology, photoacoustic imaging and its signal value (B) before and after injection of FGP NPs. (C) Autopsy HCC nodules and their corresponding pathology, photoacoustic imaging and its signal value (D) before and after injection of FGP NPs. (E) Autopsy cirrhosis nodules and their corresponding pathology, photoacoustic imaging and its signal value (F) before and after injection of FGP NPs. **Figure S7.** Photoacoustic imaging and their corresponding pathology. Take two liver lobes from each mouse (n = 7) as the photoacoustic imaging area, and then the detection accuracy of detecting small HCC in complex liver environment is evaluated by whether the photoacoustic imaging area matches the corresponding pathological examination. Among them, the photoacoustic imaging of samples ⑫ and ⑭ did not match the pathology, and the other samples were matched. **Figure S8.** Toxicity evaluated by histological analysis and its Pathoglycemia of FGP NPs. (A) HE stained sections of major organs of mice after treatment with PBS, FP NPs (0.8 mg/ml), FGP NPs (0.8 mg/ml). Scale bar, 100 μm. (B) Blood glucose level of mice in time of day. Mice (n = 3) was intravenously injected of PBS or FGP NPs at 9:00, respectively.
