# Supplementary material for: Targeted-detection and sequential-treatment of small hepatocellular carcinoma in the complex liver environment by GPC-3-targeted nanoparticles
Source: J Nanobiotechnology. 2022 Mar 24;20:156. doi: 10.1186/s12951-022-01378-w (PMC8944070; doi:10.1186/s12951-022-01378-w)
Supplement: Supplementary file 1 — Additional file 1: Figure S1. Characterization of probe. (A) BJH desorption Dv/DW pore volume of mesoporous Fe3O4 NPs. (B) stability of Fe3O4 NPs (0.4 mg/ml) measured for 7 days. Inset: A photo of Fe3O4 NPs in various solutions (from left to right: water, saline, DMEM cell medium and serum). (C) Fourier transform infrared spectra of Fe3O4 and Fe3O4-Peptide. Figure S2. Pathology of Human HCC tissues. H&E staining sections of different human HCC tissue groups (blank, Fe3O4 NPs, FP NPs). Blank: saline, FP NPs: Fe3O4-peptide nanoparticles. Figure S3. probe in human hepatic carcinoma cells. (A) Cell viability of LM3. (B) Cell viability of THLE-3. (C) Fluorescence microscope images of Calcein AM/PI co-stained with different probes (Fe3O4 NPs, FG NPs) at different concentration in acidic environment (pH = 6.5) and neutral environment (pH = 7.4). FG NPs: Fe3O4-GOD nanoparticles. Figure S4. MRI metabolism of the probe in Carcinogen-induced mice models. (A) Comparison of the liver tissues before and at 24 h after injection of FGP NPs at different time points (2, 6, 10, 14, 18, 20 weeks) during liver cancer development. Red-dotted circles represent liver nodules. (B) H&E staining sections of liver nodules of 14-week induced mice and 18-week induced mice detected by MRI. (C) In the contrast of 2-week induced mice ultrasound, 14-week induced mice with liver cirrhosis showed obvious portal vein expansion on ultrasound. And the autopsy of which showed obvious portal vein expansion. (D) Liver function and kidney function of normal mice (control), 8-week induced mice and 12-week induced mice. Figure S5. Ultrasound and 3D-Photoacoustic imaging of the process of inducing liver cancer formation. (A) Ultrasound of at different timepoints (2w, 6w, 10w, 14w, 18w, 20w) of the process of inducing liver cancer formation. (B) 3D-Photoacoustic imaging at different stages (2w, 6w, 10w, 14w, 18w, 20w) of the process of inducing liver cancer formation. Figure S6. Photoacoustic imaging and its sig [file 12951_2022_1378_MOESM1_ESM.docx]

**Additional file 1**

**Targeted-detection and sequential-treatment of small** **hepatocellular carcinoma in the complex liver environment by GPC-3-targeted nanoparticles**

Han Deng^1, 2 #^, Wenting Shang^2 #^, Kun Wang^2^, Kunxiong Guo^1,2^, Yu Liu^2^, Jie Tian^1，2, 3^ *, Chihua Fang^1, 4^ *

1. Department of Hepatobiliary Surgery, Zhujiang Hospital, Southern Medical University, Guangzhou 510280, China.

2. CAS Key Laboratory of Molecular Imaging, Beijing Key Laboratory of Molecular Imaging, the State Key Laboratory of Management and Control for Complex Systems, Institute of Automation, Chinese Academy of Sciences, Beijing, 100190, China

3. Beijing Advanced Innovation Center for Big Data-Based Precision Medicine, School of Medicine and Engineering, Beihang University, Beijing, 100191, China

4. Provincial Clinical and Engineering Center of Digital Medicine, Guangzhou, 510280, China

# These authors contributed equally to this work

* Corresponding author. Email: jie.tian@ia.ac.cn (J. Tian); zjyyfangchihua@smu.edu.cn (C. Fang)


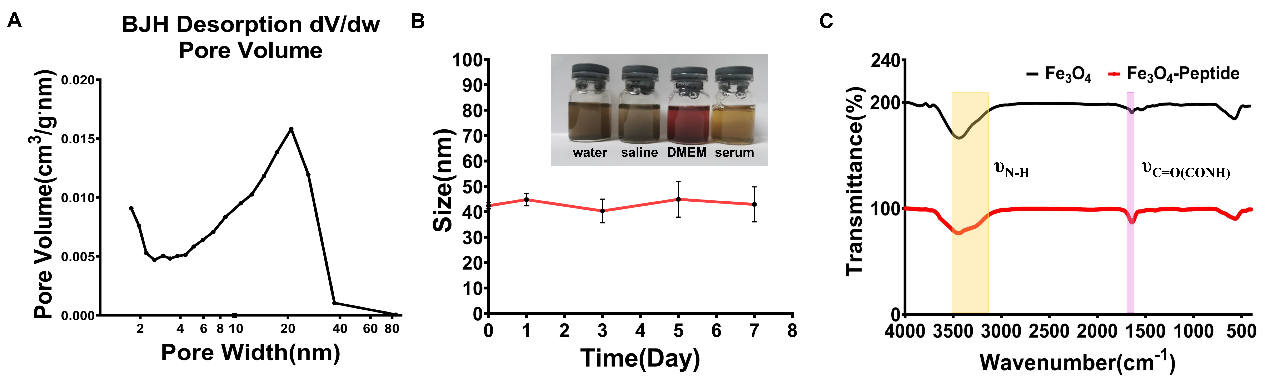


**Figure S1.** **Characterization of probe.** (A) BJH desorption Dv/DW pore volume of mesoporous Fe_3_O_4_ NPs. (B) stability of Fe_3_O_4_ NPs (0.4 mg/ml) measured for 7 days. Inset: A photo of Fe_3_O_4_ NPs in various solutions (from left to right: water, saline, DMEM cell medium and serum). (C) Fourier transform infrared spectra of Fe_3_O_4_ and Fe_3_O_4_-Peptide.


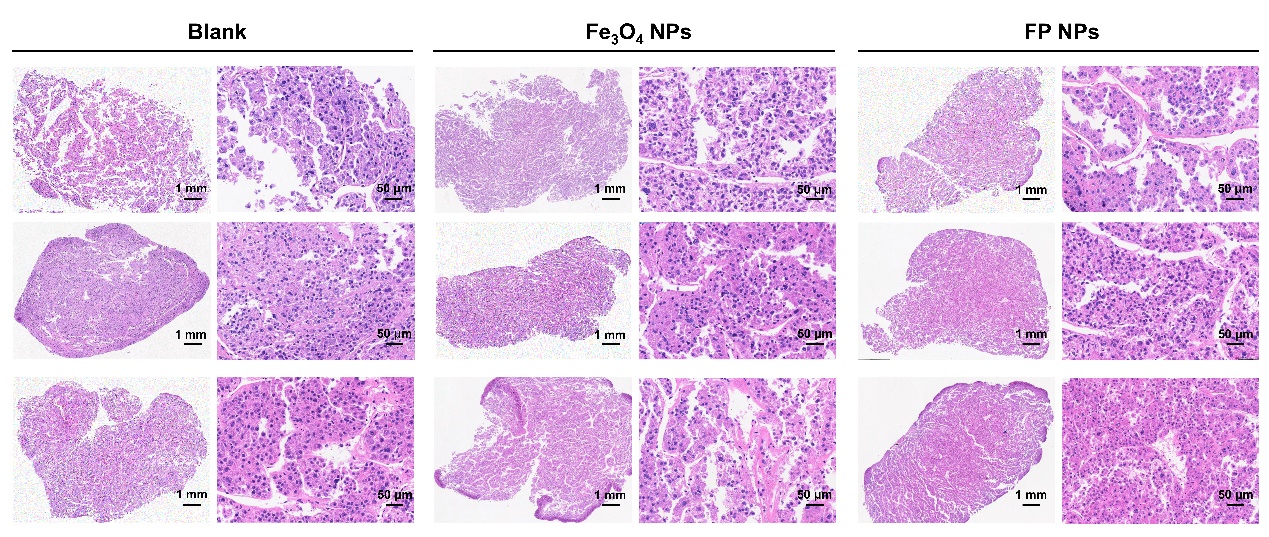


**Figure S2. Pathology of Human HCC tissues.** H&E staining sections of different human HCC tissue groups (blank, Fe_3_O_4_ NPs, FP NPs). Blank: saline, FP NPs: Fe_3_O_4_-peptide nanoparticles.


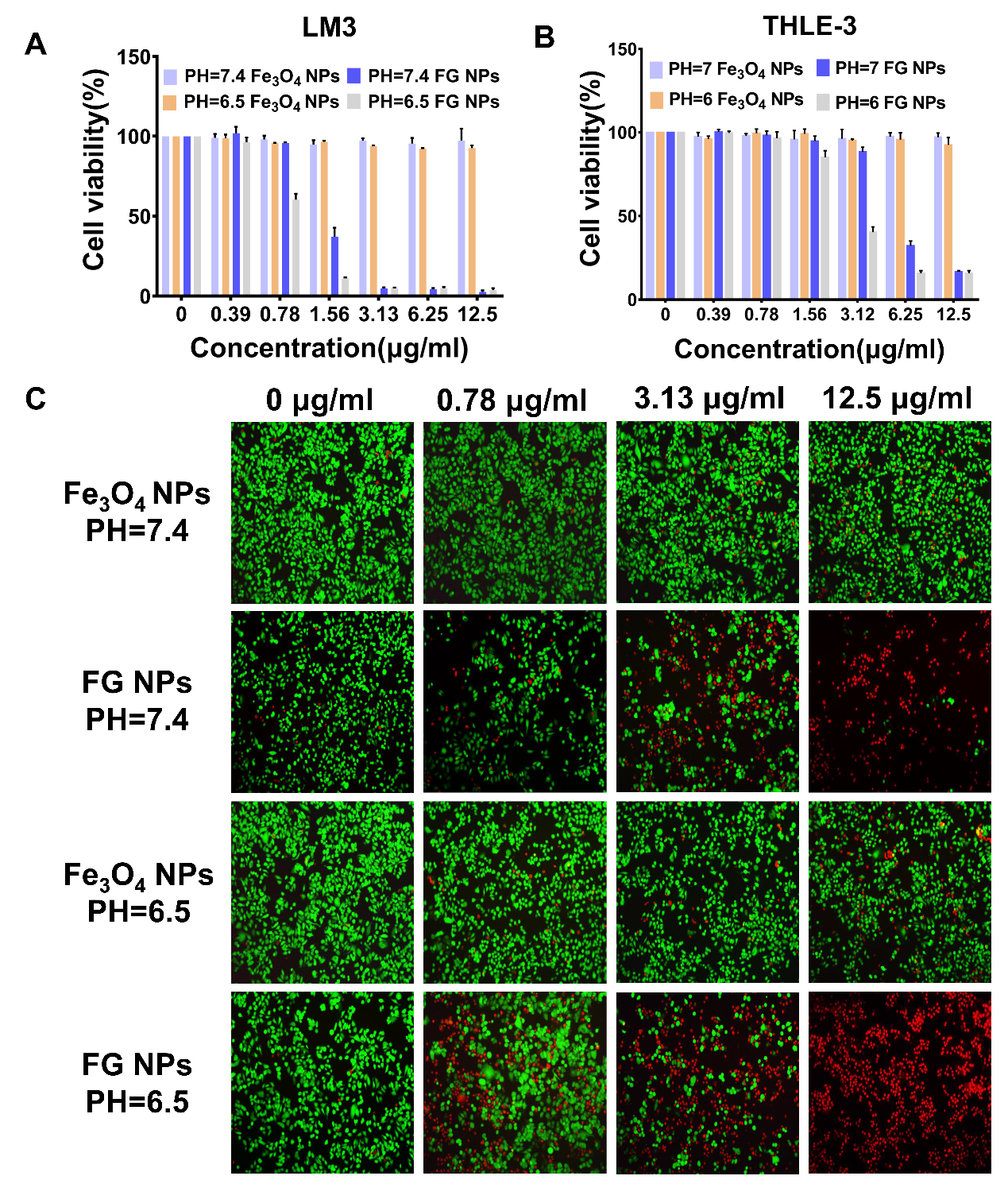


**Figure S3.** **probe in human hepatic carcinoma cells.** (A) Cell viability of LM3. (B) Cell viability of THLE-3. (C) Fluorescence microscope images of Calcein AM/PI co-stained with different probes (Fe_3_O_4_ NPs, FG NPs) at different concentration in acidic environment (pH=6.5) and neutral environment (pH=7.4). FG NPs: Fe_3_O_4_-GOD nanoparticles.


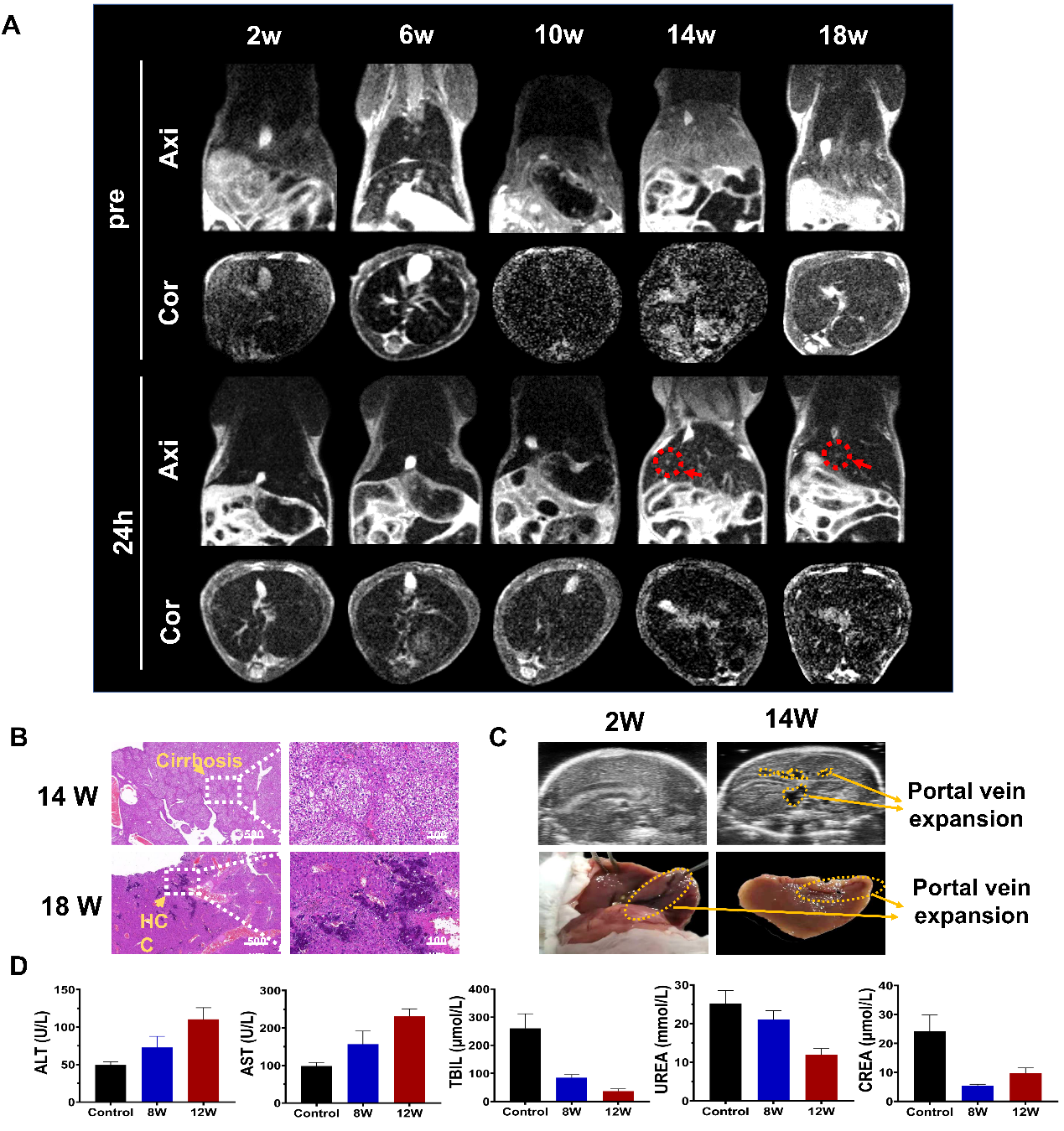


**Figure S4.** **MRI metabolism of the probe in Carcinogen-****induced mice models.** (A) Comparison of the liver tissues before and at 24 h after injection of FGP NPs at different time points (2, 6, 10, 14, 18, 20 weeks) during liver cancer development. Red-dotted circles represent liver nodules. (B) H&E staining sections of liver nodules of 14-week induced mice and 18-week induced mice detected by MRI. (C) In the contrast of 2-week induced mice ultrasound, 14-week induced mice with liver cirrhosis showed obvious portal vein expansion on ultrasound. And the autopsy of which showed obvious portal vein expansion. (D) liver function and kidney function of normal mice (control), 8-week induced mice and 12-week induced mice.


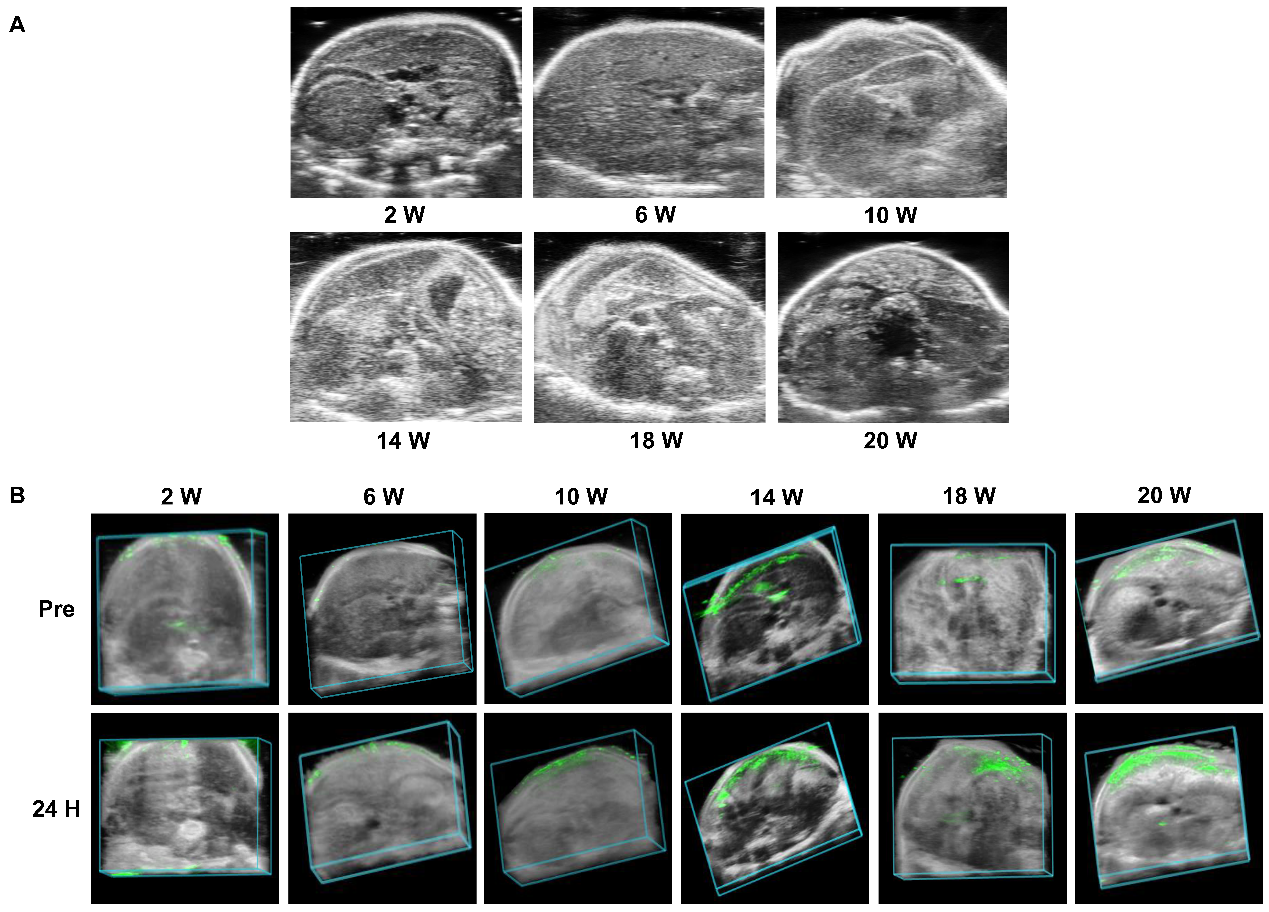


**Figure S5.** **Ultrasound and 3D-Photoacoustic imaging of the process of inducing liver cancer formation.** (A) Ultrasound of at different timepoints (2w, 6w, 10w, 14w, 18w, 20w) of the process of inducing liver cancer formation. (B) 3D-Photoacoustic imaging at different stages (2w, 6w, 10w, 14w, 18w, 20w) of the process of inducing liver cancer formation.


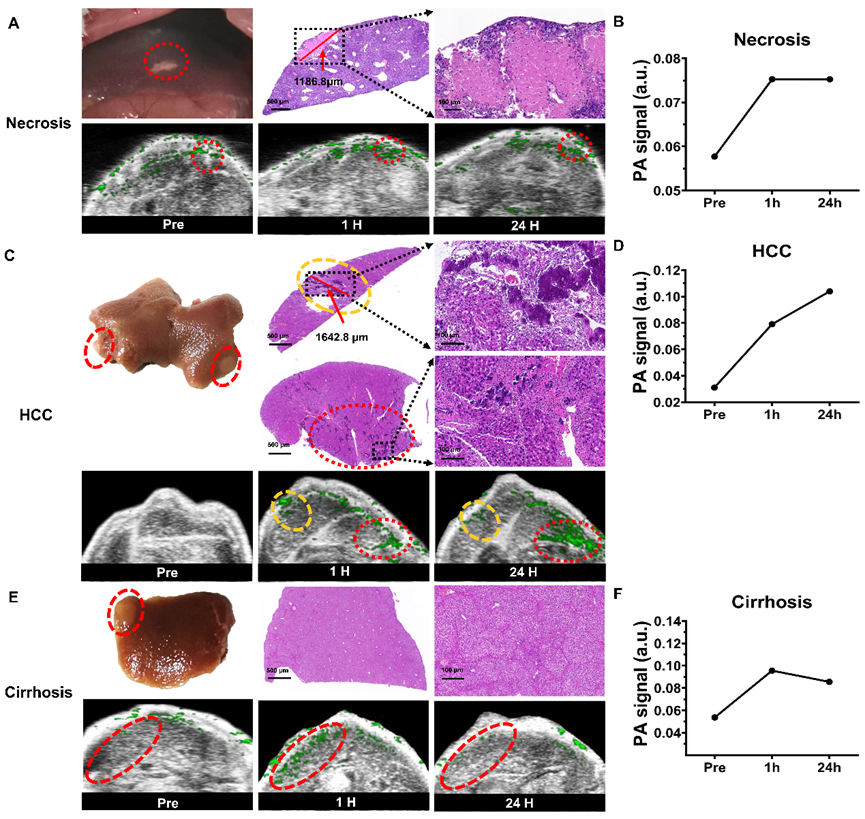


**Figure S6. Photoacoustic imaging and its signal value of necrosis nodule, HCC nodule and cirrhosis nodule.** (A) Autopsy liver necrosis nodules and their corresponding pathology, photoacoustic imaging and its signal value (B) before and after injection of FGP NPs. (C) Autopsy HCC nodules and their corresponding pathology, photoacoustic imaging and its signal value (D) before and after injection of FGP NPs. (E) Autopsy cirrhosis nodules and their corresponding pathology, photoacoustic imaging and its signal value (F) before and after injection of FGP NPs.


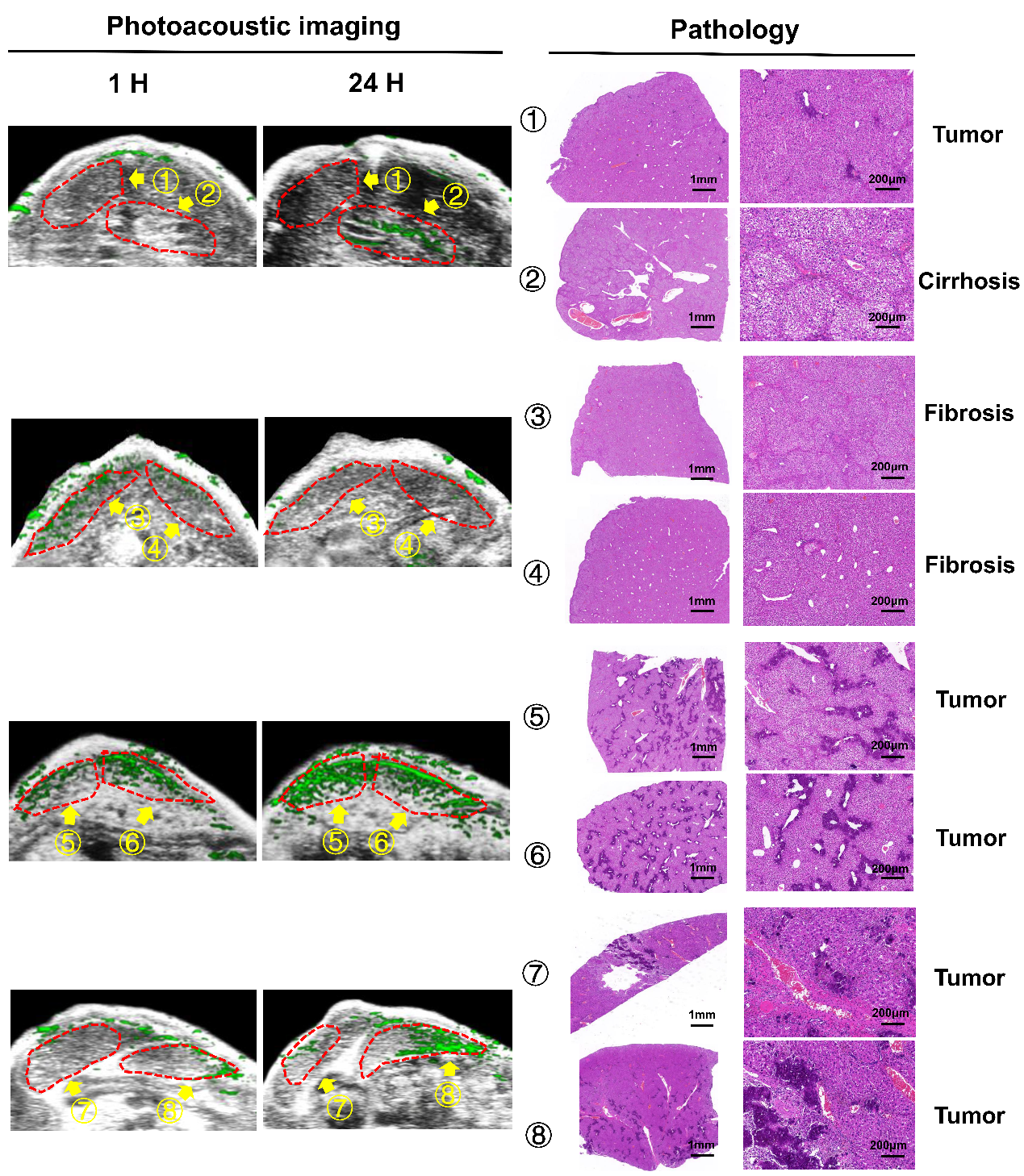


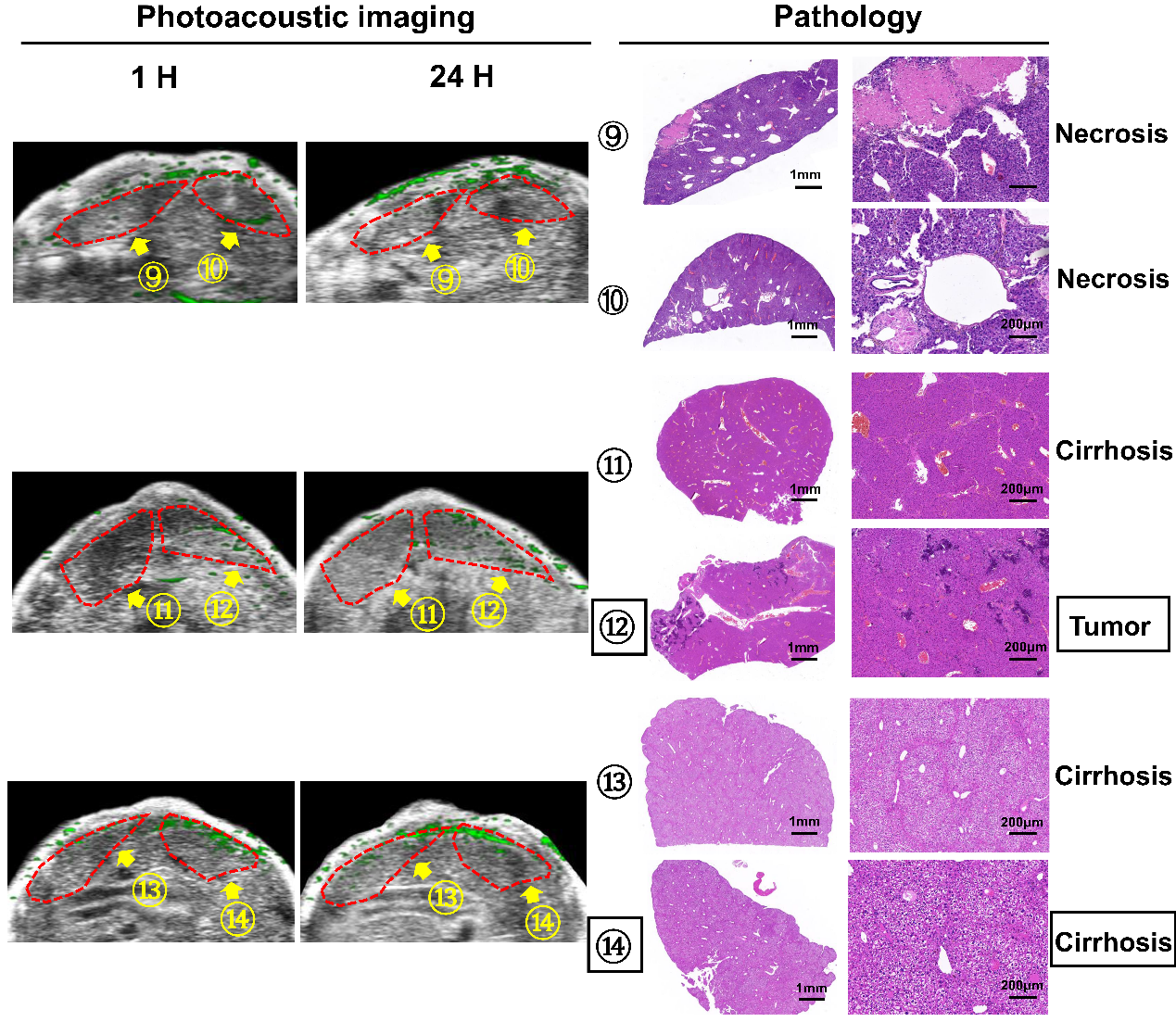


**Figure S7.** **Photoacoustic imaging and their corresponding** **pathology.** Take two liver lobes from each mouse (n=7) as the photoacoustic imaging area, and then the detection accuracy of detecting small HCC in complex liver environment is evaluated by whether the photoacoustic imaging area matches the corresponding pathological examination. Among them, the photoacoustic imaging of samples ⑫ and ⑭ did not match the pathology, and the other samples were matched.


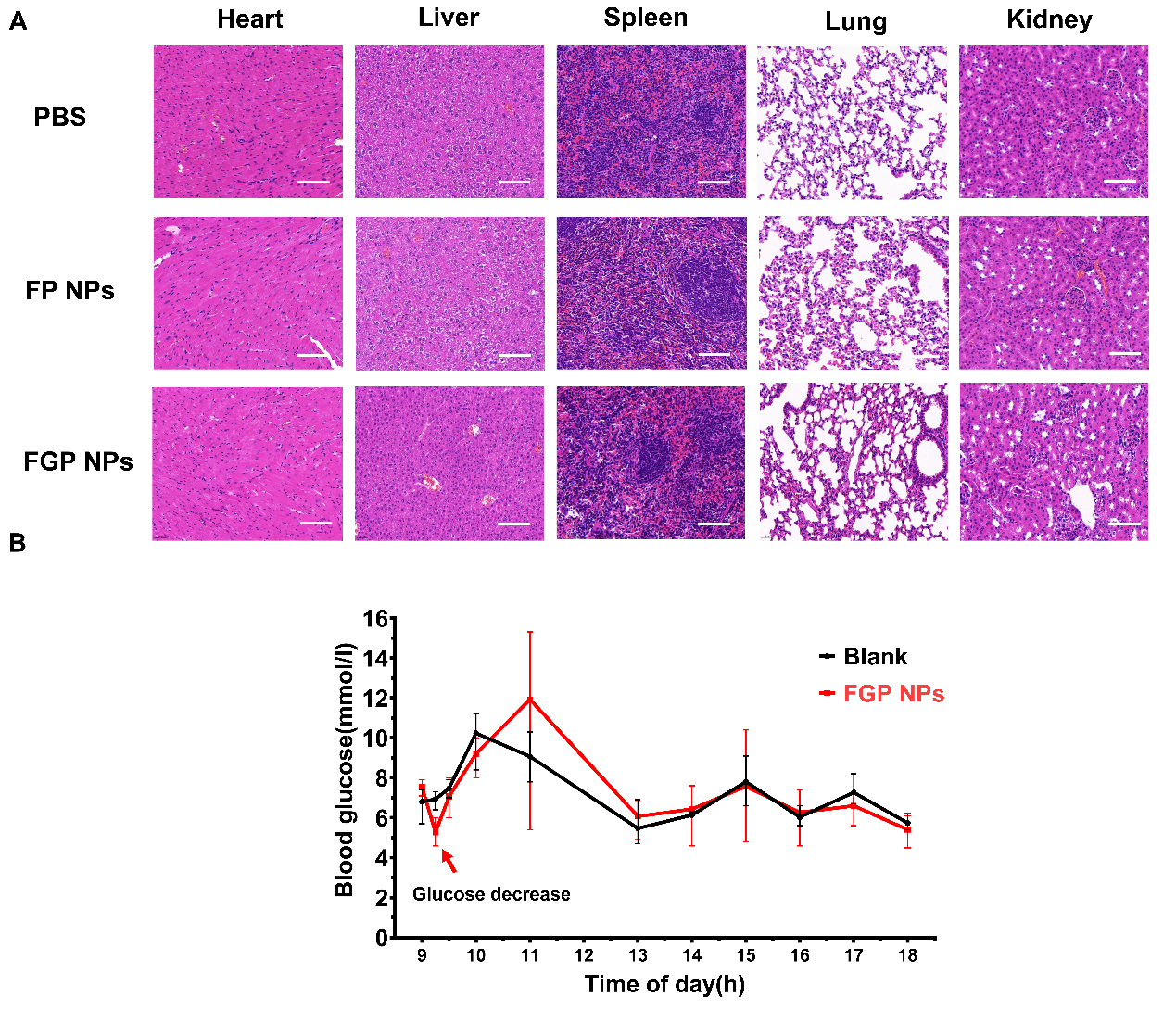
 **Figure S8.** **Toxicity evaluated by histological analysis and its Pathoglycemia of FGP NPs.** (A) HE stained sections of major organs of mice after treatment with PBS, FP NPs (0.8 mg/ml), FGP NPs (0.8 mg/ml). Scale bar, 100μm. (B) Blood glucose level of mice in time of day. Mice (n=3) was intravenously injected of PBS or FGP NPs at 9:00, respectively.
